# *Escherichia coli* Ribosomal Protein S1 Unfolds Structured mRNAs Onto the Ribosome for Active Translation Initiation

**DOI:** 10.1371/journal.pbio.1001731

**Published:** 2013-12-10

**Authors:** Mélodie Duval, Alexey Korepanov, Olivier Fuchsbauer, Pierre Fechter, Andrea Haller, Attilio Fabbretti, Laurence Choulier, Ronald Micura, Bruno P. Klaholz, Pascale Romby, Mathias Springer, Stefano Marzi

**Affiliations:** 1 Architecture et Réactivité de l'ARN, Université de Strasbourg, Institut de Biologie Moléculaire et Cellulaire-CNRS, Strasbourg, France; 2 CNRS UPR9073, University Paris Diderot, Sorbonne Paris Cité, Institut de Biologie Physico-Chimique, Paris, France; 3 Institute of Protein Research, Russian Academy of Sciences, Pushchino, Russia; 4 Institute of Organic Chemistry and Center for Molecular Biosciences, Leopold Franzens University, Innsbruck, Austria; 5 Laboratory of Genetics, Department of Biology MCA, University of Camerino, Camerino, Italy; 6 CNRS UMR 7213, Université de Strasbourg, Faculté de pharmacie, Illkirch, France; 7 Department of Integrated Structural Biology, Institute of Genetics and of Molecular and Cellular Biology, UMR 7104-CNRS, U964-INSERM, Illkirch, France; and Université de Strasbourg, Strasbourg, France; School of Medicine, University of California, United States of America

## Abstract

Regulation of translation initiation is well appropriate to adapt cell growth in response to stress and environmental changes. Many bacterial mRNAs adopt structures in their 5′ untranslated regions that modulate the accessibility of the 30S ribosomal subunit. Structured mRNAs interact with the 30S in a two-step process where the docking of a folded mRNA precedes an accommodation step. Here, we used a combination of experimental approaches *in vitro* (kinetic of mRNA unfolding and binding experiments to analyze mRNA–protein or mRNA–ribosome complexes, toeprinting assays to follow the formation of ribosomal initiation complexes) and *in vivo* (genetic) to monitor the action of ribosomal protein S1 on the initiation of structured and regulated mRNAs. We demonstrate that r-protein S1 endows the 30S with an RNA chaperone activity that is essential for the docking and the unfolding of structured mRNAs, and for the correct positioning of the initiation codon inside the decoding channel. The first three OB-fold domains of S1 retain all its activities (mRNA and 30S binding, RNA melting activity) on the 30S subunit. S1 is not required for all mRNAs and acts differently on mRNAs according to the signals present at their 5′ ends. This work shows that S1 confers to the ribosome dynamic properties to initiate translation of a large set of mRNAs with diverse structural features.

## Abstract 2

This study reveals novel insights into how *Escherichia coli* ribosomal protein S1 functions as an RNA chaperone on the ribosome, unfolding and positioning mRNAs for translation initiation.

## Author Summary

Gene expression is regulated at multiple levels, including the decision of whether or not to translate a mRNA. This phenomenon, known as translational regulation, allows rapid changes in cellular concentrations of proteins and is well suited to the adjustment of cellular growth in response to stress and environmental changes. Many bacterial mRNAs adopt structures in their 5′ untranslated regions that modulate the accessibility of the mRNA to the small ribosomal 30S subunit and so are directly involved in this regulatory process. Structured mRNAs must interact with the 30S subunit in a two-step pathway whereby the docking of a folded mRNA is followed by an accommodation step that involves unfolding of these structures. However, it is not known how the ribosome unfolds mRNA structures to promote translation initiation, nor which ribosomal factors are responsible for this activity. We demonstrate that the first three domains of ribosomal protein S1 endow the 30S subunit with an RNA chaperone activity that is essential for the binding and unfolding of structured mRNAs, allowing the correct positioning of the initiation codon for translation. However, ribosomal protein S1 is not required for all mRNAs and acts differently depending on the type of regulatory elements present in a given mRNA. In all, we have shown that ribosomal protein S1 provides an RNA-melting activity to the exit site of the 30S decoding channel and confers some plasticity on the ribosome to initiate translation of mRNAs.

## Introduction

Translation initiation, which ensures the formation of the first codon–anticodon interaction into the peptidyl (P)-site of the small ribosomal subunit in the correct frame, is the rate-limiting step of protein synthesis. Binding of the mRNA to the 30S subunit takes place at any time during the assembly of the 30S initiation complex (30SIC) and the kinetics is independent of the initiation factors, relying uniquely on 30S and mRNA features (e.g., [1],[2]). Crystal structures of the ribosome containing an unstructured mRNA and tRNA showed the mRNA path, refered as the mRNA channel, occupied by around 30 unpaired nucleotides forming numerous interactions with the 30S subunit [3]–[5]. The formation of a short duplex between the Shine–Dalgarno (SD) sequence of the mRNA and the 3′ end of the 16S rRNA (aSD) locks precisely the 5′ end of mRNA at the exit site of the mRNA channel, a specific place of the 30S known as the platform [3],[6]. The SD/aSD interaction is sufficient for unstructured model mRNAs to bind to the 30S, however most of the natural mRNAs contain additional sequences or structure motifs in their 5′ untranslated regions (UTRs) that have been exploited by bacteria to regulate translation initiation [7]–[11]. Structures in the 5′UTR of mRNAs are thought to represent a kinetic barrier that could lower translation initiation rates because the 30S must disrupt first the structures it encounters in the ribosome binding site (RBS) to allow the mRNA to reach its decoding site [2]. Several studies have revealed that mRNA structure motifs located upstream of the initiation codon bind to the 30S in a two-step process [2],[12]–[14]. A typical example is *Escherichia coli rpsO* mRNA encoding ribosomal protein (r-protein) S15, which carries a pseudoknot structure within the RBS, and which is recognized by the 30S for translation and by S15 for autoregulation [15]. Structure analysis of several ribosomal complexes identified intermediates of the initiation pathway of *rpsO* mRNA [13]. It revealed that the pseudoknot structure is first docked on the 30S platform where it forms the SD/aSD helix and interacts with r-proteins S2, S7, S11, and S18. In a second step, the pseudoknot unfolds to promote the formation of the codon–anticodon interaction at the P-site. This activity is carried out by the ribosome, but the mechanism is yet unknown.

Recent studies have shown that the 30S is endowed with an RNA helicase activity at the mRNA entry site. This helicase activity is due to the r-proteins S3, S4, and S5, which unwind mRNA structures during translation elongation [16],[17]. Are both the extremities of the mRNA channel endowed with a similar RNA unfolding activity? In other words, is the platform of the 30S able to unfold mRNA structures to promote mRNA accommodation during translation initiation? The protein environment of the 30S platform consists of several essential r-proteins, namely S1, S2, S7, S11, and S18 [5],[13],[18]. Among these proteins, S1 is an atypical r-protein because it is the largest and most acidic one that is weakly and not always associated with the 30S subunit [19]. The protein consists of six imperfect OB-fold repeats, which is an RNA-binding module specific for single-stranded regions, and is found in many proteins involved in RNA metabolism [20]. Although the structure of the protein has not yet been solved, a cryo-EM analysis suggested that the protein may adopt an elongated shape on the 30S and may bind 11 nts upstream of the SD of a model RNA [18],[21]. *E. coli* r-protein S1 is essential for the translation of many mRNAs and for viability [22]. Particularly, S1 forms an essential component of the mRNA binding site for mRNAs lacking or bearing weak SD sequences [23]–[26]. Furthermore, isolated S1 is able to melt RNA duplexes or helices independently from the 30S [27]–[31]. These works led to the hypothesis that S1 would confer to the 30S an RNA melting activity to facilitate translation of structured mRNAs, although these studies were not carried out on S1 bound to the ribosome and with natural mRNAs. Finally, S1 has been implicated in many other functions [20]. The versatility of the RNA–S1 interaction and the existence of multiple OB-fold domains might explain the diverse biological functions of S1 outside or on the ribosome.

In the present work, we demonstrate that r-protein S1 confers to the 30S an RNA chaperone activity, which is modulated by the ribosomal environment and essential for the binding and the accommodation of structured mRNAs into the decoding channel. We have analyzed the S1 dependence on three different mRNAs from *E. coli*, which all contain specific binding sites for translational repressors located close to or within the RBSs and which are repressed at the translation initiation step by various mechanisms. Using these natural mRNAs, we show that S1 on the ribosome interacts transiently with structured mRNAs and promotes a metastable folding state to create new interactions with the 30S subunit. The melting process is slow and represents most likely the rate-limiting step of translation of structured mRNAs. In contrast, an mRNA bearing optimal SD sequence and weakly structured RBS does not need S1 to form active ribosomal initiation complex. Our study reveals the mechanism of action of r-protein S1 on natural mRNAs and how S1 modulates the activity of the 30S dependent on the mRNA context.

## Results

### S1 Acts Differently on Various mRNAs for Translation Initiation

**Figure 1:**
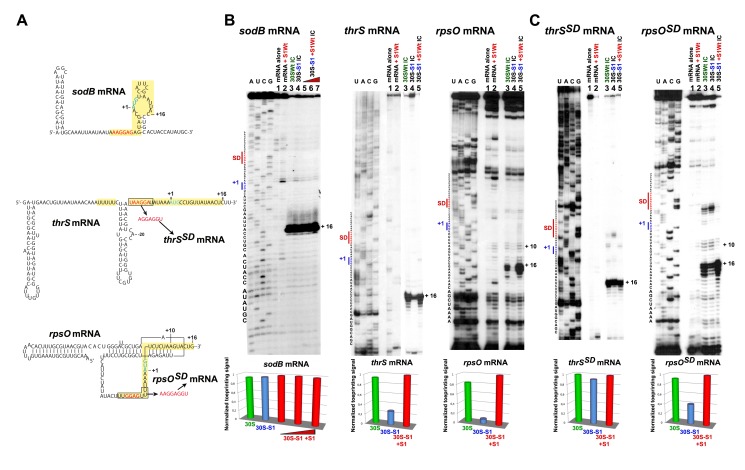
Formation of simplified initiation complexes involving three different mRNAs. (A) Secondary structure models of *sodB*, *thrS*, and *rpsO* mRNAs including their 5′UTR and the RBSs. The secondary structure of *sodB* mRNA is derived from [32], of *thrS* mRNA from [75], and of *rpsO* from [76]. The SD sequence is in red, the AUG codon in blue, and the RBS in yellow. The mutations at the SD of *thrS* and *rpsO* are specified. (B) Effect of S1 on the formation of the initiation complex (30SIC) analyzed by toeprinting assays using different mRNAs (*sodB*, *thrS*, *rpsO*). Lanes 1 and 2, incubation controls of the mRNA alone (lane 1) or bound to wild-type S1 (S1Wt, lane 2); lanes 3 and 4, the 30SIC was formed with the mRNA, the initiator tRNA, and either the wild-type 30S (lane 3, 30SWt), or the 30S lacking S1 (lane 4, 30S-S1); lanes 5–7, the 30SIC was formed with 30S-S1 pre-incubated with increasing concentrations of S1 (30S-S1+S1Wt): lane 5, 200 nM; lane 6, 500 nM; lane 7, 1 µM. Lanes A, C, G, U, sequencing ladders. Below the gels, the quantification of the toeprint was normalized according to the total amount of radioactivity (full-length extension and +16 product bands) using the SAFA software [77]. The data represented the yield of mRNA bound to 30SWt (green), 30S-S1 (blue), and 30S-S1+S1 (red). (C) Effect of S1 on the 30SIC formation analyzed by toeprinting with *thrS^SD^* and *rpsO^SD^* mRNAs, which contained an enhanced SD sequence. Same legend as in panel B.

We first monitored the effect of r-protein S1 on the formation of the 30SIC using three different natural mRNAs (Figure 1A). These mRNAs have been selected because they have evolved specific structural features to be well translated and regulated at the initiation step of translation. They also all carry an unpaired SD sequence. *E. coli sodB* mRNA (SD AAGGAG, ΔG −8.48 kcal/mol predicted for the SD/aSD helix), encoding superoxide dismutase, contains a weakly structured RBS [32] and the binding sites for the translational repressor RyhB sRNA and Hfq [33]. *E. coli thrS* mRNA (SD UAAGGA, ΔG −5.96 kcal/mol), encoding threonyl-tRNA synthetase (ThrRS), contains a bi-partite unstructured RBS interrupted by a hairpin structure recognized by ThrRS for translation repression [34]. Both RyhB and ThrRS hinder the ribosome binding to repress translation. Finally, *E. coli rpsO* mRNA (SD GGAG, ΔG −5.85 kcal/mol) contains a pseudoknot structure, which sequesters part of the coding sequence. Binding of r-protein S15 stabilizes the pseudoknot on the 30S platform to prevent the start codon from reaching the P-site [13].

Toeprinting assays were used to analyze the formation of a simplified 30SIC, composed of the 30S, the mRNA, and the initiator tRNA [35]. A toeprint is observed at position +16 (+1 is the adenine of the start codon) if the mRNA occupies the decoding channel and if the codon–anticodon interaction takes place at the P-site. To monitor the action of S1, the assays were performed with wild-type (WT) 30S, S1-depleted 30S (30S^−S1^) (Figure S1A), or the 30S^−S1^ complemented with purified r-protein S1 (30S^+S1^). Quantification of the data showed that the 30S efficiently recognizes and accommodates *sodB* mRNA into the decoding channel in the presence or in the absence of S1 (Figure 1B). Thus, S1 is dispensable for mRNA carrying an unstructured RBS with a strong SD sequence. Conversely, the formation of the 30SIC performed with the 30S^−S1^, *thrS* mRNA, or *rpsO* mRNA are strongly perturbed, showing that S1 has a role for activating these mRNAs (Figure 1B).

Because *thrS* and *rpsO* mRNAs have a weak SD, we addressed the question of whether S1 would be required for the docking and/or for the accommodation process of these mRNAs by introducing an enhanced SD (AGGAGGU, ΔG −12.53 kcal/mol) to reduce the S1 dependence for mRNA docking. Translation of *thrS^SD^* and *rpsO^SD^* mRNAs was indeed significantly enhanced *in vivo* [36],[37]. Formation of the 30SIC with *thrS^SD^* mRNA was similar with WT 30S and 30S^−S1^, indicating that S1 becomes dispensable (Figure 1C). However, for *rpsO^SD^* mRNA, the yield of 30SIC was still low when formed with 30S^−S1^. Concomitantly, several other reverse transcriptase (RT) pauses in *rpsO^SD^* mRNA were observed when WT 30S and 30S^−S1^ were bound to the mRNA. These stops located at positions −5 and +10 correspond to the entrance of the SD/aSD helix and to the pseudoknot structure, respectively (Figure 1C). They represent signatures of the stalled 30S pre-Initiation complex (30S-preIC) in which *rpsO^SD^* mRNA binds to the 30S but remains folded onto the 30S platform [13].

We then performed filter binding assays to monitor the direct binding of 5′ end-labeled mRNAs to WT 30S or 30S^−S1^ in the absence of the initiator tRNA—that is, before the accommodation step. The binding saturation curves show that S1 strongly enhances the docking of WT *thrS* and *rpsO* mRNAs on the 30S, while *sodB*, *thrS* ^SD^, and *rpsO* ^SD^ mRNAs bind to the 30S independently of S1 (Figure S1B). The three WT mRNAs bind the WT 30S (containing S1) with a similar Kd value (around 1 µM), although the SD sequence of *sodB* mRNA is stronger than the SD sequence of *thrS* and *rpsO* mRNAs. However, the absence of S1 on the 30S strongly decreases the recognition of WT *thrS* and *rpsO* mRNAs (Figure S1B). This S1-specific effect was completely alleviated when the SD was enhanced in *thrS* ^SD^ and *rpsO* ^SD^ mRNAs, and the binding affinity for the 30S increased 5-fold (Figure S1B). Therefore, a strong SD sequence compensates the lack of r-protein S1 to anchor the mRNAs onto the 30S subunit. However, the ability of *rpsO* ^SD^ mRNA to bind the 30S independently of S1 is not sufficient for its translation because S1 is still required to promote the formation of the active 30SIC as evidenced by the toeprinting assays (Figure 1C). Hence, these data indicate that S1 is directly involved in the accommodation of *rpsO* mRNA into the decoding channel.

Together, the data show that two S1 functions can be distinguished: (i) promotion of mRNA binding and (ii) mRNA accommodation. The various activities of r-protein S1 reflect the diversity of RBS architectures. The data support the following schemes where mRNAs with weakly structured RBSs (i.e., *sodB* and *thrS*) form 30SICs in a single step—that is, binding directly leads to active 30SIC formation. Instead, with structured mRNA (i.e., *rpsO*) two distinct steps have been identified, where mRNA binding (influenced by their SDs) precedes its accommodation into the decoding channel. In both cases, the need of S1 for the binding is exclusively dictated by the strength of the SD sequence, whereas S1 is essential for the accommodation of structured mRNAs.

### S1 Melts the Pseudoknot of *rpsO* mRNA on the 30S

**Figure 2:**
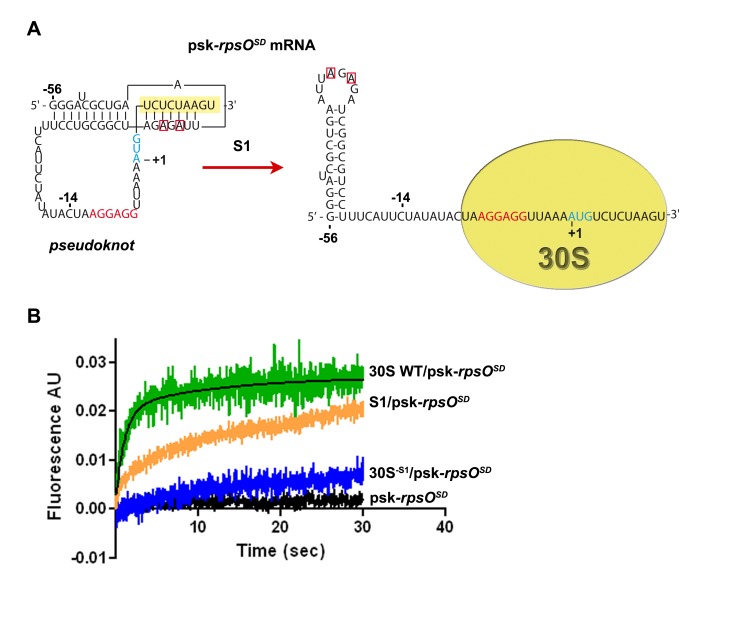
Ribosomal protein S1 induces melting of *rpsO* pseudoknot structure. (A) Two 2-APs were introduced at positions A-40 and A-42 of the pseudoknot structure of *rpsO^SD^* mRNA (psk-*rpsO^SD^*) carrying an enhanced SD sequence (nucleotides squared in red). In the pseudoknot structure, the two adenines form Watson–Crick base pairs with residues of the coding sequence (in yellow). When the mRNA is placed in the 30S decoding channel, the pseudoknot is melted and these adenines become accessible. The initiation codon AUG is in blue. (B) The spectra show the 2-AP fluorescence emission of the corresponding modified adenines upon injection of wild-type 30S (30S WT/psk-*rpsO^SD^*, in green), of 30S lacking S1 (30S^−S1^/psk-*rpsO^SD^*, in blue), or of S1 alone (S1/psk-*rpsO^SD^*, in orange). The fitting of the experimental curves was performed with graphpad PRISM software.

Because our data suggest that r-protein S1 promotes the accommodation of *rpsO* mRNA on the 30S that would require unfolding of its pseudoknot structure, we used fast kinetics to analyze the structural changes of the pseudoknot on the ribosome using 2-aminopurine (2-AP) modifications. The fluorescent nucleobase 2-AP, which can interact with uracil in a Watson–Crick pair or with cytosine in a wobble pair, is known to quench its fluorescence emission in a quantifiable manner [38], depending on local changes of the RNA structure when it stacks with other bases while fluorescence increases when it is fully exposed to solvent [39],[40]. Two modifications were introduced in a mRNA fragment encompassing the *rpsO* pseudoknot called psk-*rpsO^SD^* (containing nucleotides −56 to +12) at the strategic positions A-40 and A-42 involved in long-range interactions of the pseudoknot structure (Figure 2A). Melting of the pseudoknot on the 30S is expected to enhance fluorescence due to an increased accessibility of A-40 and A-42 towards the solvent. The kinetic of the pseudoknot melting on the 30S was analyzed by stopped-flow fluorescence experiments. The formation and stabilization of the pseudoknot structure was evidenced during the renaturation process. The addition of Mg^2+^, known to greatly stabilize the pseudoknot structure [15], causes significant quenching of the fluorescence (Figure S2A). To analyze the effect of S1 isolated or 30S-bound, we added either S1 alone, 30S containing S1, or 30S^−S1^. The time course of the increase in 2-AP fluorescence as the result of the pseudoknot melting was reproducibly observed when the RNA was incubated with the WT 30S (Figure 2B). Conversely, the addition of 30S^−S1^ to the 2-AP modified RNA only slightly changed the fluorescence emission as compared to the controls (Figure 2B). Noteworthy, binding and toeprinting experiments showed that *rpsO^SD^* mRNA is well recognized by the 30S^−S1^ but does not form an active 30SIC (Figures 1C and S1B), demonstrating that mRNA binding to the 30S is not sufficient *per se* to change the fluorescence. Therefore, our data indicate that the increased fluorescence is mediated through S1 due to an increased accessibility of A-40 and A-42 towards the solvent. Our data are consistent with previous findings showing that G-39 and G-41 of *rpsO* were highly accessible to RNase T1 in 30SIC, while these residues were not cleaved in the stalled 30S complex where the pseudoknot structure is stabilized [41]. The analysis of the stop-flow data required fitting a double exponential function, revealing two kinetic phases for the melting of the pseudoknot structure, a fast (k_fast_ 0.9 s^−1^) and a slow (k_slow_ 0.08 s^−1^) process. The k_fast_ value corresponded to the majority of the fluorescence increase (73.3%). The addition of the initiator tRNA had no effect on the kinetics, suggesting that the S1-dependent melting process does not require the formation of the anticodon–codon interaction (unpublished data). The same experiment performed with r-protein S1 alone shows marginally enhanced fluorescence emission. Saturation could not be attained even with long recording times, so that the fitting of the S1 curves could not be performed accurately (Figure S2B–C). As a control, we demonstrate that S1 deleted of the OB-fold domains 1, 5, and 6, a mutant with impaired mRNA and 30S binding (see below), did not enhance the fluorescence emission (Figure S2B–C). These experiments show that the RNA melting activity of S1 is strongly stimulated when the protein is bound to the 30S as compared to the isolated protein, indicating that S1 is primarily acting on the 30S subunit.

Thus r-protein S1 is endowed with a 30S-stimulated melting activity that leads to the unfolding of the pseudoknot structure required for the relocation of the mRNA into the decoding channel.

### Ribosomal Protein S15 Hinders the Melting Activity of S1 to Repress *rpsO* Translation

Because ribosomal protein S15 stabilizes the pseudoknot conformation of *rpsO* onto the 30S to repress its own translation, we analyzed whether r-protein S1 interferes with the regulatory function of S15 (Figure S2D). Toeprinting reveals that in the absence of initiator tRNA, formation of the trapped ribosomal complex involving S15, WT 30S, and *rpsO* mRNA causes several RT pauses around position +10, corresponding to the entrance of the pseudoknot. Identical patterns were observed with 30S^−S1^ or 30S^+S1^, indicating that the pseudoknot is stabilized by S15 regardless the presence or not of S1 on the 30S (Figure S2D). Therefore, S1 did not affect the formation of the trapped complex, while in the absence of S15, the formation of the active 30SIC was strictly dependent on S1 (Figure 1B). These data illustrate that the mRNA unfolding activity of S1 can be counterbalanced by regulatory factors such as S15, which stabilizes the mRNA in the structured form onto the 30S platform.

### The OB-Fold Domains of S1 Are Not Equivalent for *rpsO* mRNA Recognition

**Figure 3:**
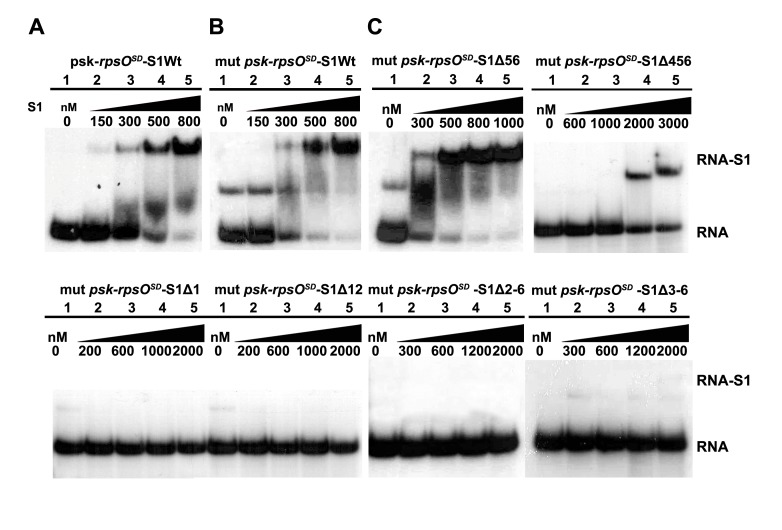
Domains 1 to 3 of r-protein S1 are essential for the recognition of *rpsO* mRNA. (A) Gel retardation assays were performed on complexes formed with the 5′ end-labeled *rpsO^SD^* mRNA (psk-*rpsO^SD^*) and wild-type r-protein S1 (S1Wt). Lane 1, incubation control of psk-*rpsO^SD^* alone; lanes 2–5, complex formation was performed with various concentrations of S1Wt as indicated on the top of the autoradiography. The addition of S1 causes fuzzy bands due to the dissociation of the complex during the migration. (B) Gel retardation assays were done on complexes formed with the 5′ end-labeled mutant C-14G psk-*rpsO^SD^* (mut psk-*rpsO^SD^*) and S1Wt. Lane 1, incubation control of mut psk-*rpsO^SD^* alone; lanes 2–5, complex formation was performed with various concentrations of S1Wt as indicated. (C) Gel retardation assays were done on complexes formed with mut psk-*rpsO^SD^* and various truncated forms of r-protein S1. The protein was deleted of either domains 5 and 6 (Δ56), domains 4 to 6 (Δ4–6), domain 1 (Δ1), domains 1 and 2 (Δ12), domains 2 to 6 (Δ2–6), or domains 3 to 6 (Δ3–6). The positions of the complex (RNA-S1) and of the free RNA (RNA) are given. Same legend as in panel B.

To gain more insight into the mechanism of interactions between S1 and the pseudoknot of *rpsO* mRNA, we analyzed deletion mutants of S1 (Table S1, Figure S3A) lacking one or more OB-fold domains based on sequence and structural information available for domains 4 and 6 [42]. To avoid possible structural heterogeneity of the *rpsO* mRNA fragment forming the pseudoknot, we also studied the mutant (C-14 to G, mut psk-*rpsO^SD^*; Figure 2A), which was shown to exclusively form the pseudoknot structure [43]. We first show that WT S1 binds similarly to the two RNAs (*rpsO^SD^* and *mut-rpsO^SD^*) and that the protein concentration around 400–500 nM causes a shift of almost 50% of the 5′ end-labeled RNAs (Figure 3A–B). These data were also well correlated with surface plasmon resonance (SPR) experiments (Kd≈350 nM; Figure S3B). The contribution of each OB-fold domain in recognizing wt or mut psk-*rpsO^SD^* mRNAs was defined using gel retardation assays (results not shown and Figure 3C, respectively). The deletion of domain 1 or of the two first N-terminal domains (Δ12) in S1 caused a complete loss of RNA binding even at a concentration of 5 µM. The removal of domains 4 to 6 (S1Δ4–6) decreased the stability by 5-fold, while the additional deletion of domain 3 (S1Δ3–6) abolished mRNA binding. Deletion of domains 5 and 6 affected RNA binding only slightly. These data correlate well with the SPR experiments, which show that the truncated protein S1Δ12 interacts weakly with psk-*rpsO^SD^* (Figure S3B). Taken together, these data demonstrate that the six OB-fold domains of S1 are not functionally equivalent, with the first three N-terminal domains of r-protein S1 being essential for the recognition of *rpsO* pseudoknot structure.

### Domains 1 to 3 of r-Protein S1 Are Essential for Cell Viability

**Figure 4:**
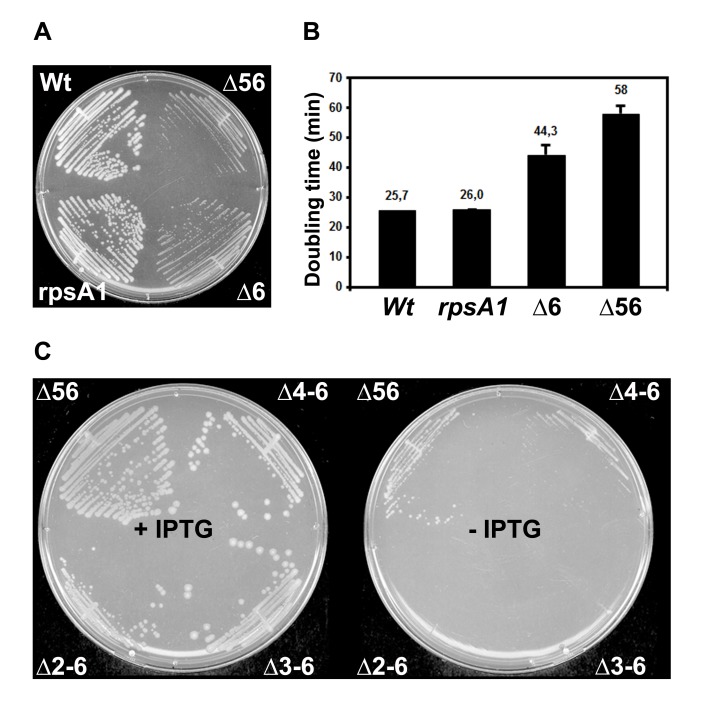
Effect of successive deletion in *rpsA* performed at the original *rpsA* locus on cell growth. (A) Growth was compared between WT strains (Wt, *rpsA1*) and strains carrying deletions of domains 6 (Δ6) and of domains 5 and 6 (Δ56) in *rpsA* on LB plates at 37°C. The *E. coli* strains are AnK02 (WT), MS77 (*rpsA1*), MS78 (Δ6), and MS79 (Δ56). (B) Measurements of the doubling times of various strains. The growth was done in LB medium at 37°C. The strains were identical to those of the panel A. (C) The growth was compared in strains carrying deletions of domains 5 and 6 (Δ56), 4 to 6 (Δ4–6), 3 to 6 (Δ3–6), 2 to 6 (Δ2–6) in *rpsA*. They were complemented with the plasmid pNK34, which carries WT *rpsA* under the control of the hybrid *trc* promoter with the *lac* operator. The experiments were done in the presence of IPTG (+IPTG) or in the absence of IPTG (−IPTG). Strains are MS79pNK34 (Δ56), MS84pNK34 (Δ4–6), MS83pNK34 (Δ3–6), and MS82pNK34 (Δ2–6).

Because the domains of S1 are not equivalent for RNA binding, we then analyzed the importance of each OB-fold domain for cell growth *in vivo*. We constructed a set of strains with the chromosomal copy of *rpsA* (the gene for S1) carrying deletions of increasing length as well as a control allele with the *kan* cassette inserted downstream of WT *rpsA*, called *rpsA1* (Figure S4A). The growth of the control strain and the levels of S1 were identical to that of the WT strain (Figure 4A–B). Two other mutant alleles carry either deletion of domain 6 (*rpsA*Δ*6*) or of domains 5 and 6 (*rpsA*Δ*56*). The alleles *rpsA1*, *rpsA*Δ*6*, and *rpsA*Δ*56* were obtained with high yields as haploids, indicating that they are viable (Figure S4B), although the growth of the two mutant strains was slower than the WT strains (Figure 4B). In addition, *rpsA*Δ*6* and *rpsA*Δ*56* alleles confer a cold-sensitive phenotype (Figure S4C). Larger replacements such as *rpsA*Δ*4–6* (deletion of domains 4 to 6), *rpsA*Δ*3–6* (deletion of domains 3 to 6), *and rpsA*Δ*2–6* (deletion of domains 2 to 6) were only obtained as diploids carrying both the WT and the mutant copy of *rpsA* (Figure S4D). We then transduced these three mutant alleles to strains transformed with the complementing plasmid pNK34, which carries the *rpsA* gene under the control of an IPTG-inducible promoter. In the absence of IPTG, the strain carrying *rpsA*Δ*4–6* was able to grow, whereas the strains carrying the larger deletions (*rpsA*Δ*3–6* and *rpsA*Δ*2–6*) did not grow, indicating that they are lethal alleles (Figure 4C). In summary, the *in vivo* experiments showed that the successive deletions of the OB-fold domains gradually affect cell growth: the two last C-terminal domains are dispensable, the additional deletion of domain 4 still allows growth but at extremely slow rates, and the further deletion of domain 3 causes lethality.

### Domains 1 to 3 of S1 Are Essential for Structured mRNA Docking and Accommodation on the 30S

**Figure 5:**
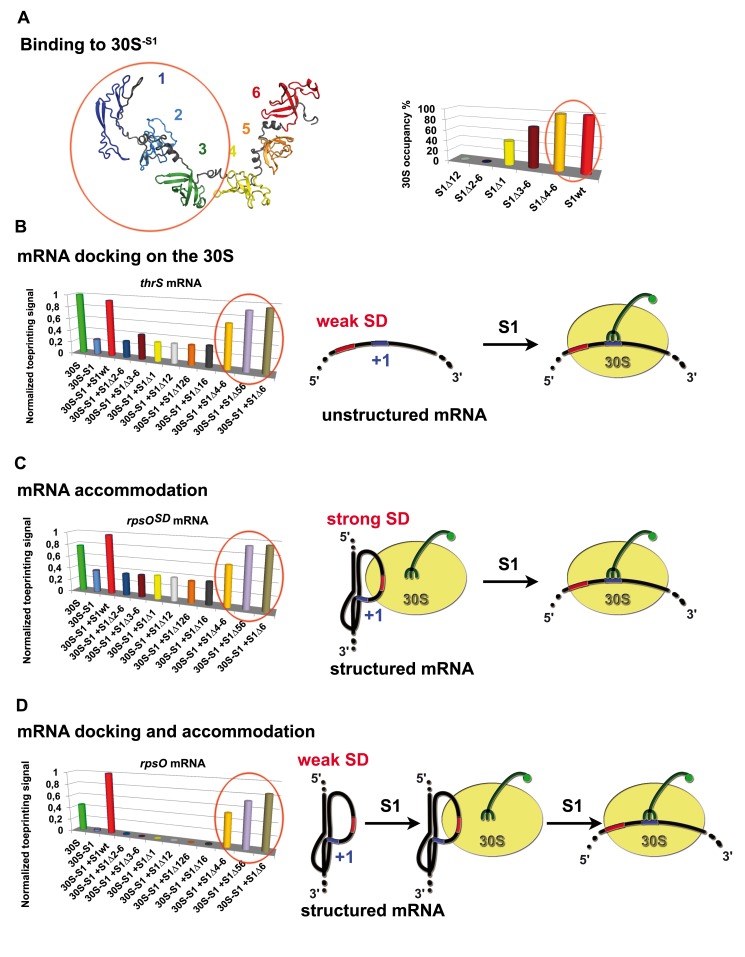
Effect of S1 variants on 30S binding and formation of the simplified initiation complex. (A) Domains 1 to 3 of r-protein S1 are required for efficient 30S^−S1^ binding. (Left panel) A model of r-protein S1 was built based on the structure of domains 4 to 6 analyzed by NMR and SAXS experiments [42],[61]. Each OB-fold domains are represented in different colors. (Right panel) Direct binding of r-protein S1 variants to 30S was visualized by Western blot analysis and quantified (see Text S1). Wild-type S1 (S1wt); S1 was deleted of domains 1 and 2 (S1Δ12), of domains 2 to 6 (S1Δ2–6), of domain 1 (S1Δ1), of domains 3 to 6 (S1Δ3–6), and of domains 4 to 6 (S1Δ4–6). (B) Domains 1 to 3 of S1 are essential for *thrS* mRNA docking on the 30S. Formation of the 30S initiation complex (30SIC) with *thrS* mRNA was probed by toeprinting. The toeprint at position +16 was quantified and normalized to the full-length RNA. The 30SIC was done with the 30S, with the 30S-S1 lacking S1, and with the 30S-S1 complemented with either wild-type S1 (S1Wt) or with the truncated forms of S1: deletion of domain 1 (S1Δ1), domains 1 and 2 (S1Δ12), domains 1, 2, and 6 (S1Δ126), domains 1 and 6 (S1Δ16), domains 5 and 6 (S1Δ56), domain 6 (S1Δ6), domains 4 to 6 (S1Δ4–6), domains 3 to 6 (S1Δ3–6), and domains 2 to 6 (S1Δ2–6). (C) Toeprinting assays performed with *rpsO^SD^* mRNA show that domains 1 to 3 of S1 are required to accommodate mRNA into the decoding channel. (D) Toeprinting assays performed with *rpsO* mRNA demonstrate that domains 1 to 3 of S1 are essentiel for the docking and the accommodation steps. (C and D) The legends are as in panel B. (B–D) A schematic drawing illustrates the key roles of S1 in the different steps of the formation of the 30SIC involving *thrS*, *rpsO^SD^*, and *rpsO* mRNAs. The 30S is colored in yellow, and the initiator tRNA is in green. The SD sequence and the AUG codon are colored in red and blue, respectively.

Because some of the domains of r-protein S1 were dispensable *in vivo*, we analyzed the implication of each OB-fold domain in binding to the 30S. The WT and mutant proteins were incubated with the 30S at a ratio of 3∶1, and the excess was removed by size exclusion chromatography. The S1-occupancy of the 30S was quantified by Western blot and revealed that a minimal protein containing domains 1 to 3 fully retains 30S binding (Figure 5A). Only the deletion of the two first N-terminal domains (1 and 2) totally abolishes 30S binding.

Formation of the active 30SIC using *thrS*, *rpsO*, or *rpsO^SD^* mRNAs, the initiator tRNA, and the 30S^−S1^ pre-incubated with the different S1 variants was monitored by toeprinting (Figures 5B–D and S5). For *thrS* mRNA, which bind the 30S in a single step process and for which unfolding is not necessary, the domains 1 to 3 of S1 are essential and sufficient to promote the formation of the active 30SIC (Figures 5B and S5B). Indeed, 70% of the 30SIC is formed with S1Δ4–6, whereas the additional deletion of domain 3 causes a strong reduction to 40%. Thus, the ability of S1 to stimulate the binding step of *thrS* mRNA is sustained by the three first N- terminal domains of S1. Similar data were obtained for *rpsO^SD^* (enhanced SD) and *rpsO* mRNAs (Figures 5C–D and S5A and S5C) where the structure of the pseudoknot needs to be unfolded on the 30S to be positioned into the decoding channel. The deletion of domains 5 and 6 only slightly affect the formation of 30SIC, while the additional deletion of domain 4 decreases the 30SIC yields to 50% and 60% for *rpsO* and *rpsO^SD^*, respectively. The removal of domains 3 to 6 completely abolished the formation of 30SIC for *rpsO* mRNA, whereas a residual signal of 30% was observed for *rpsO^SD^*. The enhanced SD compensates the lack of S1 for the binding step as demonstrated by filter binding assays (Figure S1B), but a minimal core of S1 (domain 1–3) is still important to promote the unfolding/accommodation second step. Noteworthy, S1Δ3–6 binds efficiently to the 30S but with impaired functions, suggesting that domains 1 to 3 are essential for all the steps including the binding of *thrS* and *rpsO* mRNAs, and the accommodation of *rpsO* mRNA. All in all, these data show that both 30S-dependent activities of S1, the docking of mRNA carrying weak SD (as for *thrS* and *rpsO*) and the unfolding of structured mRNA and accommodation into the mRNA channel (as for *rpsO* and *rpsO^SD^*), require the first three OB-fold domains of r-protein S1. Hence, domains 1 to 3 constitute the minimal protein that retains most of the S1 functions with respect to structured mRNAs.

## Discussion

### Ribosomal Protein S1 Unfolds Structured mRNAs on the Ribosome

The ability of isolated r-protein S1 to unwind model RNA duplexes or helices has been well documented [2],[27]–[31]. However, it was not yet demonstrated that S1 would be the key r-protein to unfold mRNA structures on the ribosome. In this study, we have monitored the action of r-protein S1 on the natural structured and regulated *E. coli rpsO* mRNA encoding r- protein S15 during the formation of the 30SIC. This mRNA carries a pseudoknot structure within the RBS, which is recognized by the 30S for translation [15]. It sequesters the beginning of the coding sequence through base pairings that need to be melted for the formation of the codon–anticodon interaction [13],[15].

**Figure 6:**
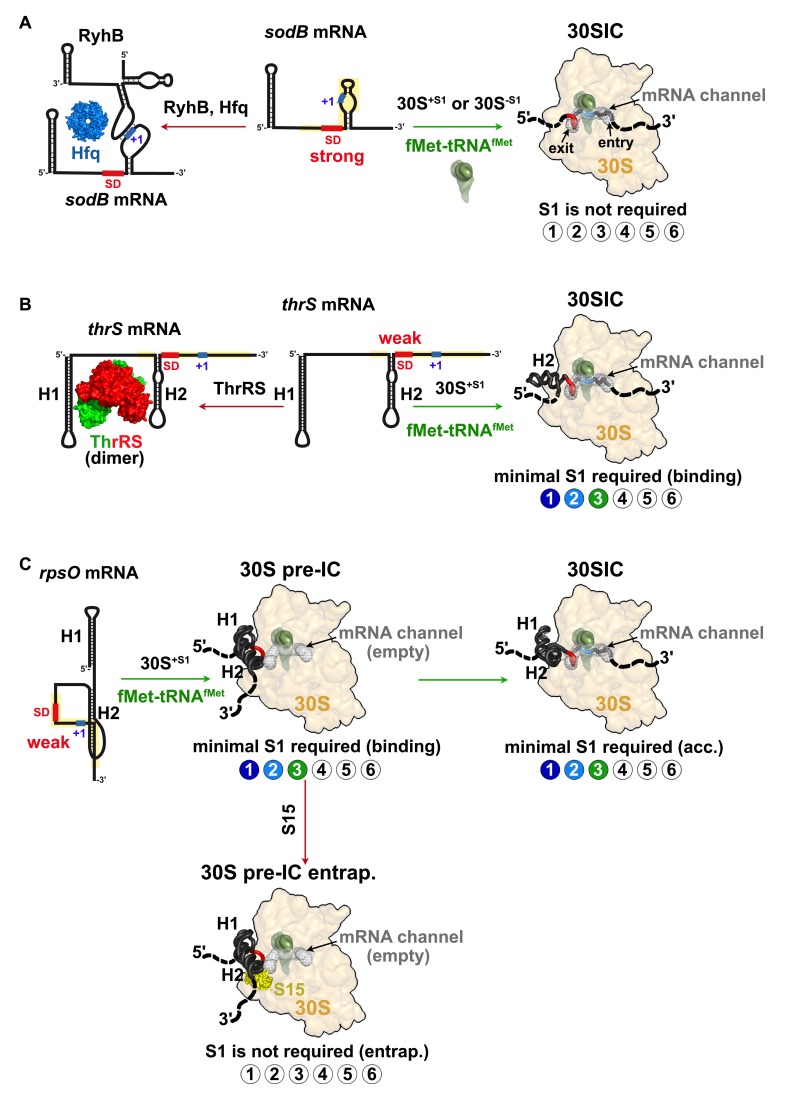
Different mechanisms of action of S1 r-protein. (A) Formation of the 30S initiation complex (30SIC) with *E. coli sodB* mRNA is S1-independent. The mRNA contains a weakly structured RBS (in yellow) and a strong SD sequence (in red) [32]. Under iron depletion, binding of RyhB and Hfq to *sodB* mRNA occludes the 30S binding and causes rapid degradation of the mRNA [78],[79]. (B) A single step pathway to form the initiation complex with *thrS* mRNA. The bipartite RBS comprised an unstructured SD sequence and a U-rich sequence upstream of the H2 domain. The three first domains of S1 are essential and sufficient for the docking of *thrS* mRNA to the 30S. The free ThrRS binds to the H1 and H2 hairpin motifs to prevent the 30S binding (adapted from [34]). (C) A two-step pathway to form the 30SIC involving *E. coli rpsO* mRNA. The pseudoknot structure is recognized by the 30S and by r-protein S15 (in yellow). Domains 1 to 3 of S1 are required both for the docking and the accommodation of *rpsO* mRNA on the 30S to relocate the initiation codon into the P-site. The free S15 binds and stabilizes the pseudoknot on the 30S and prevents the initiation codon to reach the decoding center. The six OB-fold domains of S1 are schematized by circles: colored circles are the active domains of S1, and white circles represented the domains that are not required for the translation of a given mRNA. The color code is as found in Figure 5A.

We demonstrate here that r-protein S1 and primarily its three OB-fold domains 1 to 3 are essential for the accommodation process allowing *rpsO* mRNA to unfold and to relocate its initiation codon into the decoding center. Using 2-AP–modified *rpsO* mRNA, we were able to follow the S1-dependent melting of the pseudoknot directly on the ribosome. We could also compare the S1 RNA melting activity isolated or on the ribosome (Figure 2). Using a combination of approaches, we show that the fluorescence emission does not result from the interaction of the mRNA on the 30S but is primarily due to the melting of the pseudoknot structure (Figures 2A and 6C). In its natural ribosomal context, the melting activity of S1 is clearly more pronounced and is independent of the presence of the initiator tRNA. This enhanced activity on the 30S could be explained by different conformations of S1 when free in solution or anchored to the ribosome where the OB-fold domains 1 to 3 would be orientated in an optimal way to interact with *rpsO* mRNA. An alternative explanation is the possible contribution of other ribosomal components to the S1-dependent unfolding process.

A recent single-molecule study demonstrated that isolated r-protein S1 is able to melt in a multistep process a large artificial 274 bp stem-loop structure by binding to an upstream single-stranded RNA region [31]. This elegant study showed that S1 binds to the transient open form of the helix-unpaired junction region and stabilizes the open form to promote the local melting of the base pairs. This model is consistent with our data that are obtained on a natural structured mRNA. We propose that the three first domains of S1 bind successively to the A/U-rich connecting loop next to the long-range interaction allowing S1 to bind to the transiently opened base pairs. This mechanism would then lead to pseudoknot unwinding. The rate (0.9 s^−1^) at which the pseudoknot conformational change takes place on the 30S is rather slow as compared to the rates determined for other events of the translation initiation pathway [1]. It could thus represent the rate-limiting step of the initiation of structured mRNA as it was previously proposed [44]. Our data also indicate that the initiator tRNA is not essential for the RNA melting process. However, the formation of the anticodon–codon interaction is critical to stabilize *rpsO* mRNA into the channel of the 30S.

### The r-Protein S1 Has Various Activities Depending on the mRNA Signatures

Using three *E. coli* natural mRNAs (*sodB*, *thrS*, *rpsO*), we demonstrate that r-protein S1 acts differently according to the nature of the signals present in the 5′UTR of mRNAs to form the 30SICs. Indeed, S1 is dispensable for the formation of the initiation complex involving *sodB* mRNA, which contains a strong SD and a weakly structured RBS (Figure 6A). In the second example, S1 is required for the docking of *thrS* mRNA onto the ribosome in a single step process and the replacement of its weak SD with a stronger one alleviates the requirement of S1 for the formation of 30SIC (Figure 6B). Finally, S1 is required for the recruitment of *rpsO* mRNA through its pseudoknot structure and for the accommodation process allowing the mRNA to occupy the decoding channel (Figure 6C). Noteworthy, pseudoknots were preferentially selected as strong binders of *E. coli* ribosomes or of free r-protein S1, while SD-containing unstructured mRNAs were selected against S1-depleted 30S ribosomes [45]. Hence, the complexity of mRNA structure within the RBS would direct the choice of the S1 actions to promote the formation of active 30SIC (Figure 6). In addition, we show that the action of S1 can be prevented by repressor proteins such as r-protein S15, which binds to *rpsO* pseudoknot and prevents its melting onto the ribosome to repress translation (Figure 6). One can predict that other translational regulatory proteins would interfere with the action of S1 onto the ribosome.

This variety of mechanisms is consistent with the fact that S1 is weakly associated to the 30S subunit. In agreement with this observation, a subpopulation of ribosomes lacking S1 was suggested to co-exist in *E. coli* under normal growth conditions [46]. Furthermore, the overexpression of *rpsA* led to the dissociation of leaderless mRNAs from the ribosomes [47]. This was supported by the fact that the overproduction of S1 slightly enhanced the occupancy of the ribosomes, suggesting that the WT levels of the protein did not saturate the ribosomes [48]. Under stress conditions, subpopulations of ribosomes were recently isolated *in vivo*, which selectively translated leaderless mRNAs [49],[50]. Altogether, it is tempting to speculate that the absence of S1 on the ribosome might confer selectivity for specific mRNAs with strong SDs and unstructured RBSs, such as *sodB* mRNA or leaderless mRNAs. Thus, S1 confers to the ribosome the ability to dynamically adapt to the sequence and structure of mRNAs, increasing ribosome plasticity. This might help the ribosome to coordinate and fine-tune the rate of protein synthesis.

### The OB-Fold Domains of S1 Cooperate for RNA Binding But Carry Distinct Functions

S1 belongs to the family of RNA-binding proteins composed of multiple RNA-binding motifs. It contains six OB (oligonucleotide/oligosaccharide-binding) fold domains that are connected by short linkers (Figure 5A). We show here that these domains exhibit distinct but also synergistic functions. We first demonstrated that the two N-terminal domains are critical to anchor S1 onto the 30S subunit (Figure 5A). Numerous studies supported the localization of S1 on the 30S platform where it makes contacts with mRNAs and r-proteins [18],[51]–[58]. More precisely, domain 1 of S1 was shown to interact with the coiled-coil domain of r-protein S2 [59]. In addition, we show that domain 2 and to a much lesser extent domain 3 enhance binding of S1 to 30S (Figure 5A). This would suggest that other ribosomal components contributed to precisely position S1 on the 30S platform so that domains 4 to 6 would be exposed to the solvent to recruit specific mRNAs at the initiation step.

Domains 1 to 3 of S1 are essential and sufficient to promote the formation of active 30SIC involving either *thrS* or *rpsO*, while domain 4 exerted a stimulating effect only on *rpsO*, providing additional interactions required for full biological function. This is well correlated with the *in vivo* data since successive deletions of the OB-fold domains had an increasing effect on cell growth. Indeed, the two last C-terminal domains 5 and 6 affected growth rate in a limited way as it was previously shown [60], while the deletion of domains 4 to 6 permitted growth at extremely slow rates and any further deletions (Δ3–6, Δ2–6) caused complete lethality (Figure 4). This effect on cell growth can be explained by the fact that the truncated proteins are still able to bind to the ribosome, while the recruitment and/or the accommodation of essential mRNAs is presumably strongly perturbed. Although domain 1 has been mainly described as the 30S binding site, we show here that this first N-terminal OB-fold domain is also critical for *rpsO* mRNA binding (Figure 3C). Other studies revealed that various RNA substrates bind to the same surface area of a protein carrying domains 3 to 5 [61]. In addition, domain 3 with either domain 2 or domain 4 of S1 confer high affinity through cooperative contacts with RNAs [48]. Directed evolution of S1 to enhance translation of GC-rich mRNAs in *E. coli* selected mutations primarily in domains 3 and 4 [62]. Hence, the flexibility of the domains respective to each other might confer to S1 a high adaptability to bind a large variety of RNA substrates.

The work presented here provides the notion that the six domains of S1 are not functionally equivalent, although they are structurally related with respect to a common fold. The deletion of the two last C-terminal domains of S1 had no major effects on cell growth, indicating that they are not required for translation [60]. In addition, deletion of domain 6 did not affect the translation and autoregulation of *rpsA* [63]. However, the absence of the C-terminal domain causes a cold-sensitive phenotype most likely due to an impaired ability to melt RNA structures stabilized at low temperature. The fact that mutations could alter the chaperone activity preferentially at low temperatures is not so surprising. Indeed, S1 r-protein does not use energy like other RNA helicases, and therefore at the permissive temperature, the thermal energy may help the protein to melt RNA secondary structures. It could also be possible that domains 5 and 6 contribute to the translation of specific mRNAs as it was previously proposed [60],[64].

In conclusion, this study shows that r-protein S1 confers a chaperone activity to the 30S subunit that promotes the active docking and accommodation of structured mRNAs into the decoding channel. In addition, the data are indicative of a hierarchy of mRNA targets with respect to S1 recognition on the ribosome. Because S1 is essential in *E. coli*, phylogenetic analysis may shed light on how the S1 functions have evolved among bacteria. A phylogenetic study has been carried out on r-protein S1 based on structural signatures present within each OB-fold domain [42]. This analysis revealed that S1 from Gram-negative bacteria (proteobacteria, chlamidiae, spirochates, bacteroides, aquificae), thermotogae, chloflexi, and high G+C content Gram-positive bacteria (actinobacteria) contained at least the four first domains, suggesting that most of the activities of S1 would be preserved in these organisms. Although the actinobacteria, such as *Micrococcus luteus*, contained an additional fifth domain different from *E. coli* S1, *M. luteus* S1 was able to substitute *E. coli* S1 on the ribosome to translate mRNAs with weak SD [23],[65]. Another group of bacteria including the firmicutes, tenericutes, and cyanobacteria contained shorter forms of the protein with a first N-terminal domain that differs greatly from *E. coli* S1, questioning the ability of these proteins to bind to the ribosome. Two S1 homologues containing three OB-fold domains were identified in *Synechococcus*. One of these homologues was able to bind the ribosome and was found to be essential for the translational initiation of several mRNAs [66]. In *B. subtilis*, S1 protein is not essential [67]–[69], consistent with the fact that the protein plays no major role in translation [23],[42],[70]. In these Gram-positive bacteria, most of the mRNAs carry a strong SD sequence, and the low G+C content of their genomes may disfavor the formation of very stable mRNA structures, which might obviate the need for S1 melting activity on the 30S. Whether these truncated forms of S1 act as RNA chaperones outside the ribosome remains to be studied. It would also be of interest to analyze how the functions of S1 have evolved, and what are the strategies used by the ribosomes to translate structured mRNAs, in the low GC content Gram-positive bacteria.

## Methods

### Plasmids and Strain Constructions

All strains and plasmids, which have been used and constructed in this study, are given in Table S1; the oligonucleotides (oligos) used for cloning and for mutagenesis are given in Table S2. Experimental details for the constructions of the strains are given in the Text S1.

### RNA Preparation

WT *thrS*, *thrS^SD^* (−195 to +65 nts, +1 being the A of the *thrS* translational initiation codon), WT *rpsO* and *rpsO^SD^* (−120 to +65) transcripts were prepared *in vitro* by T7 transcription of linearized plasmids (see [36] for *thrS* and [37] for *rpsO* constructs). WT *sodB* mRNA (−55 to +64 nts) was transcribed from the PCR product on the genomic DNA of *E. coli* MG1655 using the appropriate oligonucleotides (Table S2). The *psk* and mut-*psk* (−56 to +12, +1 being the A of the *rpsO* initiation codon) RNA fragments have been transcribed from linearized plasmids (Table S1). The 5′ end-labeling of dephosphorylated RNA or of the chemically synthesized RNA was performed with T4 polynucleotide kinase and [γ-^32^P]-ATP [71]. All RNAs were purified on 8% polyacrylamide-8 M urea slab gel electrophoresis (PAGE). Before use, mRNAs were renatured as follows: incubation at 90°C for 1 min in RNase-free water, cooled in ice for 1 min, and at 25°C for 30 min in the appropriate buffer containing monovalent ions and MgCl_2_. Predictions of the SD/aSD stabilities were obtained using RNAcofold of the Vienna package [72].

### Preparation of Wild-Type and Mutant Proteins S1 and of 30S

Wild-type and mutant *rpsA* genes were cloned in vectors pET23a or pDEST14, and the plasmids were transformed into *E. coli* strain BL21 (Table S1). The proteins carrying six histidines at their C-terminus were purified using an affinity chromatography followed by a monoQ (for details, see Text S1). We have verified by mass spectrometry that S1 was homogeneous and was not contaminated by *E. coli* Hfq (see Text S1).

*E. coli* 30S subunits were purified on sucrose gradients after dissociation of the 70S [73]. Ribosomal protein S1 was removed from the 30S using a polyU-sepharose 4B column (Text S1).

### Toeprinting

The formation of a simplified translational initiation complex with mRNA (toeprinting assays) was done according to Fechter et al. [73]. Experimental details are given in Text S1.

### Fluorescence Measurements

RNA fragments (psk-*rpsO*, G-56 to U12) containing two 2-AP nucleotides (A-40 and A-42) were synthesized on Pharmacia Gene Assembler or Applied Biosystems instrumentations using *2′-O-*TOM protected phosphoramidite nucleoside building blocks [74]. All experiments were measured on a Kintek SF-2400 stopped-flow device at 37°C. The renatured psk-*rpsO^SD^* mRNA (50–100 nM) present in 20 mM Tris-HCl pH 7.5, 60 mM NH_4_Cl, 1 mM DTT, 7.5 mM MgCl_2_ was placed in one of the two syringes just before the experiment. The r-protein S1, 30S, 30S^−S1^, or 30S/fMet-tRNA (1 or 2 µM) in the same buffer were introduced in the second syringe. The protein and the 30S were incubated at 37°C for 20 min before their injection in the mixing chamber. The melting of the pseudoknot psk-rpsO^SD^ was monitored by measuring the increment of the fluorescence signal after passing the samples through KV408 filters (Schott) at 405 nm, generated by the 2-APs excited at 308 nm. The k_fast_ and k_slow_ values obtained by double exponential fitting were obtained with the Prism Graphpad software.

### Interaction of the 30S with r-Protein S1

Purified WT and mutant proteins S1 (150 pmoles) were incubated with 30S^−S1^ (50 pmoles) for 15 min at 37°C in 20 µl of 20 mM Tris-HCl pH 7.5, 60 mM KCl, 40 mM NH_4_Cl, 10 mM MgCl_2_, 3 mM DTT, and 0.02 mg/ml BSA. After purification on a Superdex 200 HR 10/30, the fractions containing the 30S or S1 were analyzed on a 4%–12% SDS-PAGE, and visualized by Western blots using antibodies against the His-tag of each S1 (Text S1).

### Gel Retardation Assays

Protein S1 was pre-incubated for 15 min at 37°C in the S1 buffer containing 20 mM Tris-HCl pH 7.5, 10 mM MgCl_2_, 60 mM KCl, 40 mM NH_4_Cl, 3 mM DTT, and 0.02 mg/ml BSA. Complex formation was performed at 37°C for 15 min with the renatured 5′ end-labeled RNA (12,000 cpm) and increasing concentrations of r-protein S1 in 10 µl of S1 buffer.

## Supporting Information

Figure S1 **Effect of r-protein S1 on the formation of 30S initiation complexes.** (A) Comparative analysis of the wild-type 30S and the 30S lacking S1 r-protein. Purified wild-type 30S (30S) and 30S lacking S1 (30S^−S1^) were analyzed on a 4%–12% polyacrylamide-SDS gel electrophoresis. Protein markers were run in parallel. The proteins were revealed after staining of the gel with brilliant blue and analyzed by mass spectrometry. Only r-protein S1 was missing in 30S^−S1^. (B) Filter binding assays monitor the interaction of *thrS*, *rpsO*, *sodB*, *thrS^SD^*, and *rpsO^SD^* mRNAs with the 30S subunits. Binding assays were carried out with wild-type 30S or 30S^−S1^ without initiator tRNA and various concentrations of 5′ end-labeled mRNA (5, 10, 20, 30, and 40 pmoles). The quantity of bound mRNA was represented as the function of the total concentration of the mRNA in the assays. Kd values have been estimated by fitting the binding curve with the Prism Graphpad software.
(JPG)

Figure S2 **The melting activity of r-protein S1 is prevented by the translational repressor r-protein S15.** (A) Effect of Mg^2+^ on the conformation of the 2-AP modified pseudoknot as followed by fluorescence stopped flow analysis. Two spectra were registered as a function of time. The assays were performed on the pseudoknot of *rpsO^SD^* (psk-*rpsO^SD^*) incubated in the buffer without Mg^2+^ (in black) or in the presence of 15 mM Mg^2+^ (in green). The renaturation process performed in the presence of Mg^2+^ induces a decrease of the fluorescence signal illustrating the stabilization of the pseudoknot structure where the two 2-APs at positions A-40 and A-42 form Watson–Crick base pairs with the coding sequence. The fitting of the curve shows that the process is rapid. (B and C) Effect of the S1 mutant S1Δ126 on the melting of the pseudoknot. The spectra show the 2-AP fluorescence emission upon injection of wild-type S1 (orange) or S1Δ126 (magenta) at different time scales (40 s for panel B and 200 s for panel C). The trace obtained with wild-type S1 (S1) showed in Figure 2 is reported in grey for comparison. (D) Effect of r-protein S1 on S15-mediated autoregulation. Gel fractionation of 5′ end-labeled DNA products obtained by primer extension with MMLV RT. Lane 1, incubation control of *rpsO* mRNA alone; lane 2, binding of *rpsO* mRNA to r-protein S15; lane 3, formation of the binary complex between *rpsO* mRNA and the 30SWt; lanes 4 and 5, formation of the 30S initiation complex (30SIC) involving *rpsO* mRNA, initiator tRNA, and 30SWT, in the absence or in the presence (+S15) of r-protein S15, respectively; lane 6, formation of the binary complex between *rpsO* mRNA and 30S^−S1^; lanes 7 and 8, formation of the 30SIC involving *rpsO* mRNA, the initiator tRNA, and 30S^−S1^, in the absence or in the presence (+S15) of r-protein S15, respectively; lane 9, formation of the binary complex between *rpsO* mRNA and 30S^−S1^ reconstituted with S1 (30S^+S1^); lanes 10 and 11, formation of the 30SIC involving *rpsO* mRNA, the initiator tRNA and 30S^+S1^, in the absence or in the presence (+S15) of S15, respectively; lanes G and A are sequencing lanes. The RT stops at position +10 correspond to the entrance of *rpsO* pseudoknot, whereas the toeprint at position +16 corresponds to the 30SIC where the codon–anticodon interaction takes place.
(JPG)

Figure S3 **The r-protein S1 mutants and effect of the mutations on RNA binding.** (A) Schematic representation of the deletion performed in *rpsA* for *in vitro* studies. On the top of the gel, the six OB-fold domains of S1 are represented with different colors. Polyacrylamide-SDS gel electrophoresis was performed on 30S, 30S^−S1^ (lacking S1), and r-protein S1. Lane 1, 30S; lane 2, 30S^−S1^ lacking S1; lane 3, wild-type S1 (S1 Wt); lane 4, deletion of domain 1 (S1Δ1); lane 5, deletion of domain 6 (S1Δ6); lane 6, deletion of domains 1 and 2 (S1Δ12); lane 7, deletion of domains 5 and 6 (S1Δ56); lane 8, deletion of domains 1, 2, and 6 (S1Δ126); lane 9, deletion of domains 4 to 6 (S1Δ4–6); lane 10, S1 deletion of domains 1 and 6 (S1Δ16); lane 11, deletion of domains 3 to 6 (S1Δ3–6); lane 12, deletion of domains 2 to 6 (S1Δ2–6). A ladder with various size markers is given. All proteins were purified to homogeneity. (B) SPR real-time sensorgrams showing dose-dependent interaction between psk-*rpsO^SD^* mRNA and various proteins. Increasing concentrations of proteins (9 nM in red, 19 nM in pink, 39 nM in orange, 78 nM in yellow, 156 nM in light green, 312 nM in dark green, 625 nM in light blue, 1,250 nM in dark blue, and 2,500 nM in purple) have been injected to the immobilized pseudoknot psk-*rpsO^SD^* mRNA (190 RU). As proteins, we used wild-type S1 (S1-WT) or S1 deleted of domain 6 (S1Δ6), of domains 5 and 6 (S1Δ56), of domains 4 to 6 (S1Δ4–6), and of domains 1 and 2 (S1Δ12). Binding curves were double-reference subtracted from buffer blank and reference flow cell (without RNA) and adjusted to the molecular weight of the proteins (Response = (RU/MW)×10,000). SPR was used to determine the K_D_ for psk-S1WT interaction by equilibrium binding measurements. The light grey insert in the top panel is a representative SPR response at equilibrium from three experiments.
(JPG)

Figure S4 **Constructions of** ***rpsA*** **mutant strains** ***in vivo*** **.** (A) Construction of the *rpsA1* allele. The *rpsA* gene (with its six domains) is shown with its proximal promoter (rightwards arrow) and its putative terminator (schematised as a stem-loop structure). The drawing is not to scale. A 926 bp long PCR DNA fragment was used to insert *kan* sequences immediately downstream of the translation termination site of *rpsA* (see Text S1 for the recombineering protocols). (B) Schematic representation of the construction of the two viable *rpsA* alleles deleted of domain 6 or domains 5 and 6. The constructs were verified on an agarose gel analysis of the PCR fragments made with the resulting strains (MS65 for Δ6 and MS64 for Δ56) in comparison to *rpsA1* (MS66) and wild-type (MG1655). The PCR reaction was performed with oligonucleotides AK68 (complementary to the junction between the domains 3 and 4) and KAV04 (complementary to sequences downstream of *rpsA*) in the sense and antisense directions, respectively (Table S2). (C) Phenotypic analysis of *rpsA* alleles deleted of domain 6 or domains 5 and 6. Strains MS78 and MS79 carrying the Δ6 and Δ56 *rpsA* alleles, respectively, and control strains carrying the WT (AnK02) and *rpsA1* (MS77) alleles were streaked on LB plates at the indicated temperatures. (D) Construction of diploid strains. Schematic representation of the constructions and verification of the constructs on an agarose gel analysis of the PCR fragments made with the resulting strains (MS63 for Δ4–6, MS62 for Δ3–6, and MS61 for Δ2–6) in comparison to WT *rpsA* (MG1655, *rpsA*+). The PCR reaction was performed with oligonucleotides KAV01 (complementary to sequences in domain 1) and KAV04 in the sense and antisense directions, respectively. (E) Measurement of the levels of the different S1 derivatives in haploid (Δ6 and Δ56) and diploid strains (Δ4–6, Δ3–6, Δ2–6). Western blot of extracts from strains carrying different alleles of *rpsA* (lanes 1 to 7) was performed using rabbit anti-S1 and anti-S2 sera (generous gifts of Prof. K. Nierhaus and Prof. I. Boni, respectively). The *in vivo* synthesised r-protein S1 variants in the different lanes are designated by black dots. Lane 8 was loaded with a mixture of purified S1 fragments. The truncated r-proteins S1 carry a tag made of 6 His residues, explaining the slightly slower migration of the purified fragments. The quantity of S1 (WT) and each of the fragments is of 15 ng. The extracts are from strains MG 1655 (WT), MS71 (*rpsA1*), MS72 (Δ6), MS73 (Δ56), MS63 (Δ4–6), MS62 (Δ3–6), and MS61 (Δ2–6). The calculations made to measure the molar quantities of each of the fragments are explained in the Text S1.
(JPG)

Figure S5 **Analysis of the formation of the 30S initiation complex (30SIC) by toeprinting assays.** (A) Toeprinting assays performed with wild-type *rpsO* mRNA (WT *rpsO*). Lane 1, incubation control of mRNA alone; lanes 2 to 11, incubation controls of mRNA in the presence of WT and the different truncated variants of r-protein S1 as indicated; lane 12, formation of 30SIC formed with WT *rpsO*, wild-type 30S (30SWt), and initiator tRNA^Met^; lane 13, formation of 30SIC formed with the 30S lacking S1 (30S^−S1^); lanes 14–23, 30SIC performed with WT *rpsO*, tRNA, and 30S^−S1^ reconstituted with WT S1 (lane 14, 30S^+S1^), or with S1 deleted of domains 2 to 6 (lane 15, S1Δ2–6), deleted of domains 3 to 6 (lane 16, S1Δ3–6), deleted of domain 1 (lane 17, S1Δ1), deleted of domains 1 and 2 (lane 18, S1Δ12), deleted of domains 1, 2, and 6 (lane 19, S1Δ126), deleted of domains 1 and 6 (lane 20, S1Δ16), deleted of domains 4, 5, and 6 (lane 21, S1Δ4–6), deleted of domains 5 and 6 (lane 22, S1Δ56), or deleted of domain 6 (lane 23, S1Δ6). Lanes U, A, C, and G, sequencing ladders. (B) Formation of the 30SIC involving *thrS* mRNA, 30S, and the initiator tRNA. Same legend as in the panel A. (C) Formation of the 30SIC involving *rpsO^SD^* mRNA, 30S, and the initiator tRNA. The mRNA carries a reinforced SD sequence. Left gel, lane 1, incubation control of mRNA alone; lanes 2 to 5, incubation controls of mRNA in the presence of WT and different truncated r-proteins S1 as indicated; lane 6, formation of the 30SIC formed with WT *rpsO*, 30SWt, and initiator tRNA^Met^; lane 7, 30SIC formed with 30S^−S1^; lanes 8–10, 30SIC formed with WT *rpsO*, the initiator tRNA, and 30S–^S1^ reconstituted with S1Δ1 (lane 8), S1Δ56 (lane 9), and S1Δ6 (lane 10). Middle gel, lane 1, incubation control of mRNA alone; lanes 3 to 7, incubation controls of mRNA in the presence of WT and different truncated r-proteins S1 as indicated; lane 8, 30SIC formed with WT *rpsO*, 30SWt, and initiator tRNA^Met^; lane 9, 30SIC formed with 30S^−S1^; lanes 10–14, 30SIC formed with WT *rpsO*, the initiator tRNA, and 30S–^S1^ reconstituted with S1Δ12 (lane 10), S1Δ126 (lane 11), S1Δ16 (lane 12), S1Δ2–6 (lane 13), and S1Δ3–6 (lane 14). Right gel, lane 1, incubation control of mRNA alone; lanes 3 to 6, incubation controls of mRNA in the presence of WT and truncated r-proteins S1 as indicated; lane 7, 30SIC formed with WT *rpsO*, 30SWt, and initiator tRNA^Met^; lane 8, 30SIC formed with 30S^−S1^; lanes 9–13, 30SIC formed with WT *rpsO*, the initiator tRNA, and 30S^−S1^ reconstituted with S1Δ4–6 (lane 9), S1Δ12 (lane 10), S1Δ126 (lane 11), S1Δ16 (lane 12). Lanes U, A, C, and G, sequencing ladders. Quantification of the data are given in the corresponding Figure 5C.
(JPG)

Table S1 **Strains and plasmids used in this study.**
(DOCX)

Table S2 **List of oligonucleotides.**
(DOCX)

Text S1 **Supplementary experimental procedures.**
(DOCX)

## Abbreviations

2-AP: 2-aminopurine
30SIC: 30S initiation complex
aSD: anti-Shine–Dalgarno sequence
IPTG: Isopropyl β-D-1-thiogalactopyranoside
mRNA: messenger RNA
nts: nucleotides
OB: oligonucleotide/oligosaccharide-binding
(P)-site: peptidyl-tRNA site of the ribosome
RBS: ribosome binding site
r-protein: ribosomal protein
RT: reverse transcriptase
SD: Shine–Dalgarno sequence
SPR: surface plasmon resonance
UTR: untranslated regions
WT: wild-type
