# Supplementary figures and images for: Escherichia coli Ribosomal Protein S1 Unfolds Structured mRNAs Onto the Ribosome for Active Translation Initiation
Source: PLoS Biol. 2013 Dec 10;11(12):e1001731. doi: 10.1371/journal.pbio.1001731 (PMC3858243; doi:10.1371/journal.pbio.1001731)

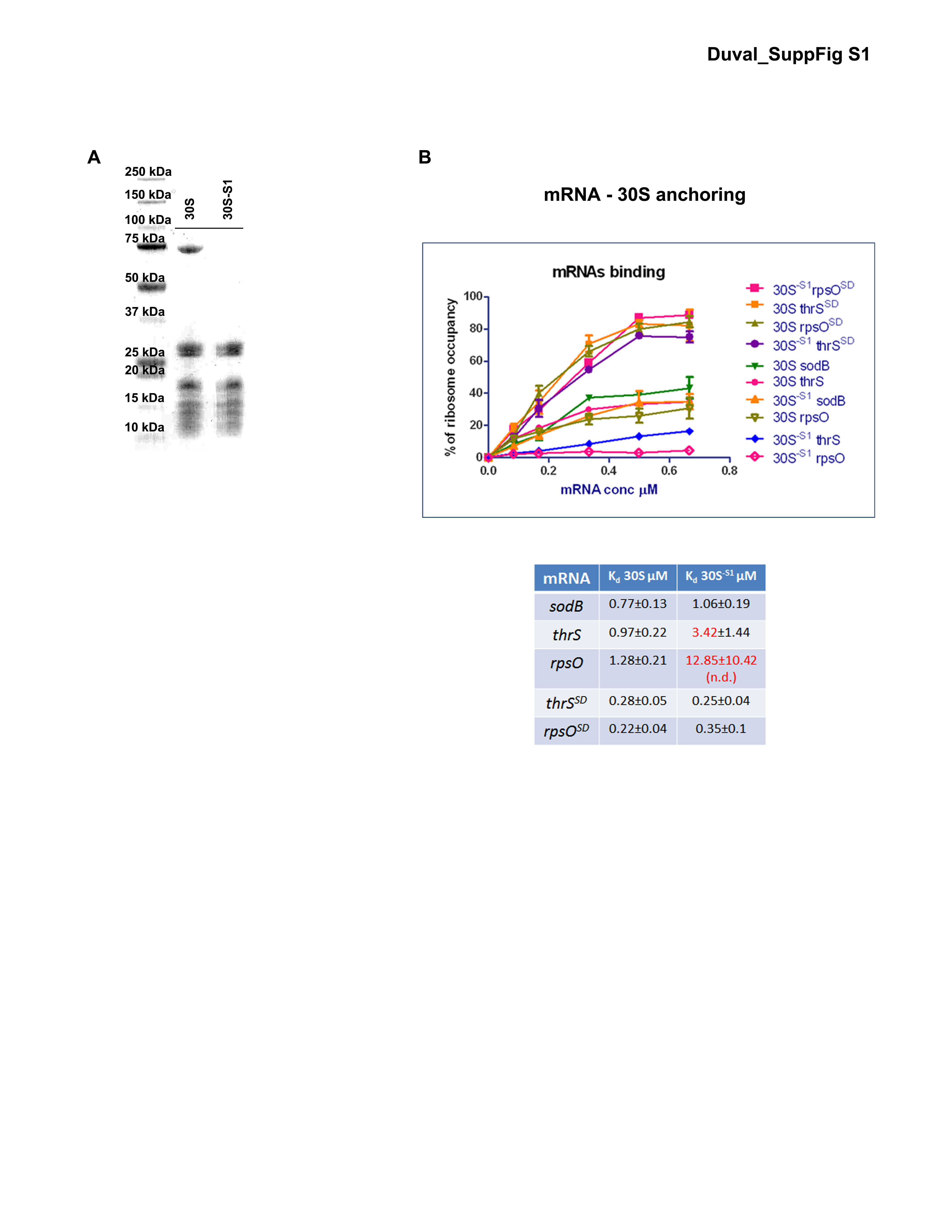

Supplement: Figure S1 — Effect of r-protein S1 on the formation of 30S initiation complexes. (A) Comparative analysis of the wild-type 30S and the 30S lacking S1 r-protein. Purified wild-type 30S (30S) and 30S lacking S1 (30S−S1) were analyzed on a 4%–12% polyacrylamide-SDS gel electrophoresis. Protein markers were run in parallel. The proteins were revealed after staining of the gel with brilliant blue and analyzed by mass spectrometry. Only r-protein S1 was missing in 30S−S1. (B) Filter binding assays monitor the interaction of thrS, rpsO, sodB, thrSSD, and rpsOSD mRNAs with the 30S subunits. Binding assays were carried out with wild-type 30S or 30S−S1 without initiator tRNA and various concentrations of 5′ end-labeled mRNA (5, 10, 20, 30, and 40 pmoles). The quantity of bound mRNA was represented as the function of the total concentration of the mRNA in the assays. Kd values have been estimated by fitting the binding curve with the Prism Graphpad software. (JPG) [file pbio.1001731.s001.jpg]

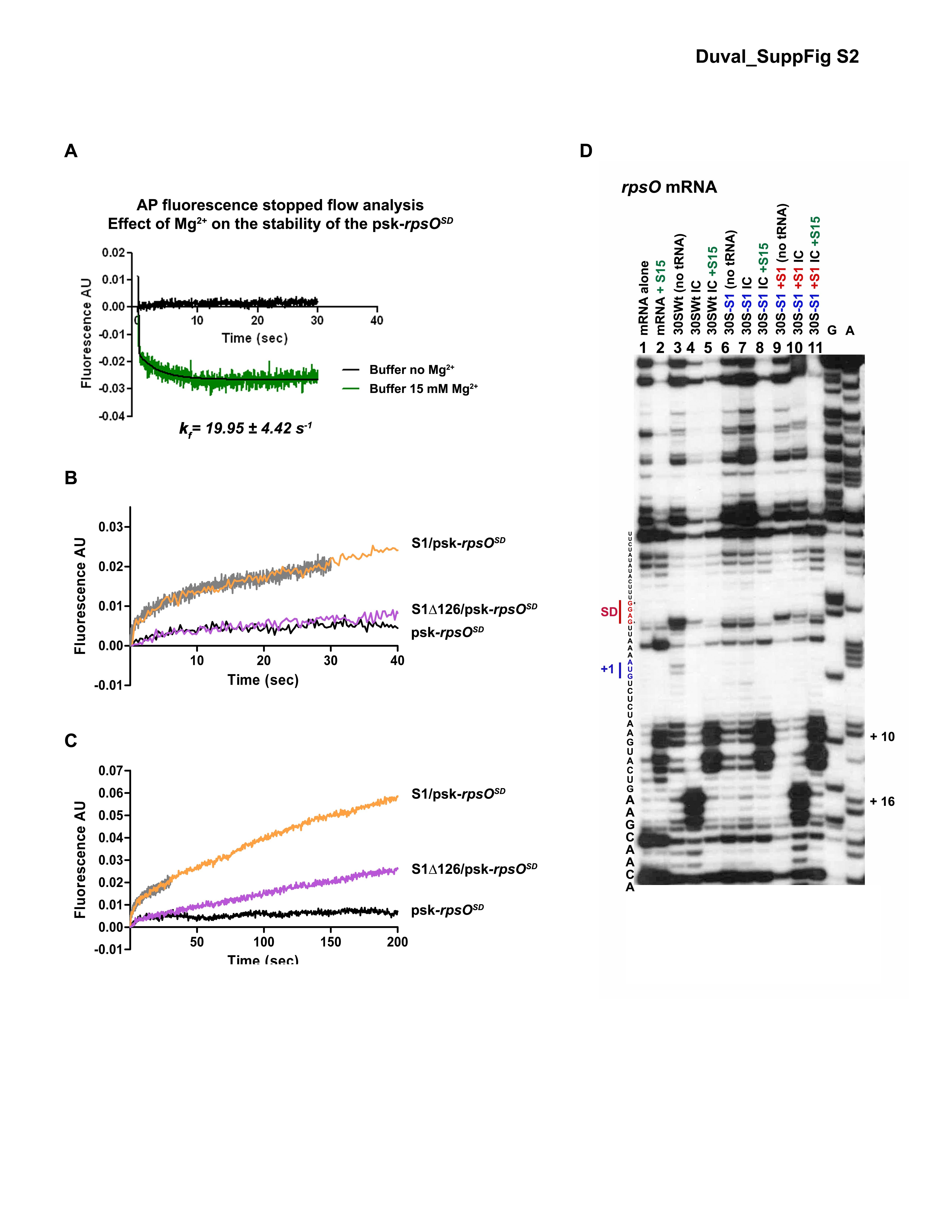

Supplement: Figure S2 — The melting activity of r-protein S1 is prevented by the translational repressor r-protein S15. (A) Effect of Mg2+ on the conformation of the 2-AP modified pseudoknot as followed by fluorescence stopped flow analysis. Two spectra were registered as a function of time. The assays were performed on the pseudoknot of rpsOSD (psk-rpsOSD) incubated in the buffer without Mg2+ (in black) or in the presence of 15 mM Mg2+ (in green). The renaturation process performed in the presence of Mg2+ induces a decrease of the fluorescence signal illustrating the stabilization of the pseudoknot structure where the two 2-APs at positions A-40 and A-42 form Watson–Crick base pairs with the coding sequence. The fitting of the curve shows that the process is rapid. (B and C) Effect of the S1 mutant S1Δ126 on the melting of the pseudoknot. The spectra show the 2-AP fluorescence emission upon injection of wild-type S1 (orange) or S1Δ126 (magenta) at different time scales (40 s for panel B and 200 s for panel C). The trace obtained with wild-type S1 (S1) showed in Figure 2 is reported in grey for comparison. (D) Effect of r-protein S1 on S15-mediated autoregulation. Gel fractionation of 5′ end-labeled DNA products obtained by primer extension with MMLV RT. Lane 1, incubation control of rpsO mRNA alone; lane 2, binding of rpsO mRNA to r-protein S15; lane 3, formation of the binary complex between rpsO mRNA and the 30SWt; lanes 4 and 5, formation of the 30S initiation complex (30SIC) involving rpsO mRNA, initiator tRNA, and 30SWT, in the absence or in the presence (+S15) of r-protein S15, respectively; lane 6, formation of the binary complex between rpsO mRNA and 30S−S1; lanes 7 and 8, formation of the 30SIC involving rpsO mRNA, the initiator tRNA, and 30S−S1, in the absence or in the presence (+S15) of r-protein S15, respectively; lane 9, formation of the binary complex between rpsO mRNA and 30S−S1 reconstituted with S1 (30S+S1); lanes 10 and 11, formation of the 30SIC involving rpsO mRNA, th [file pbio.1001731.s002.jpg]

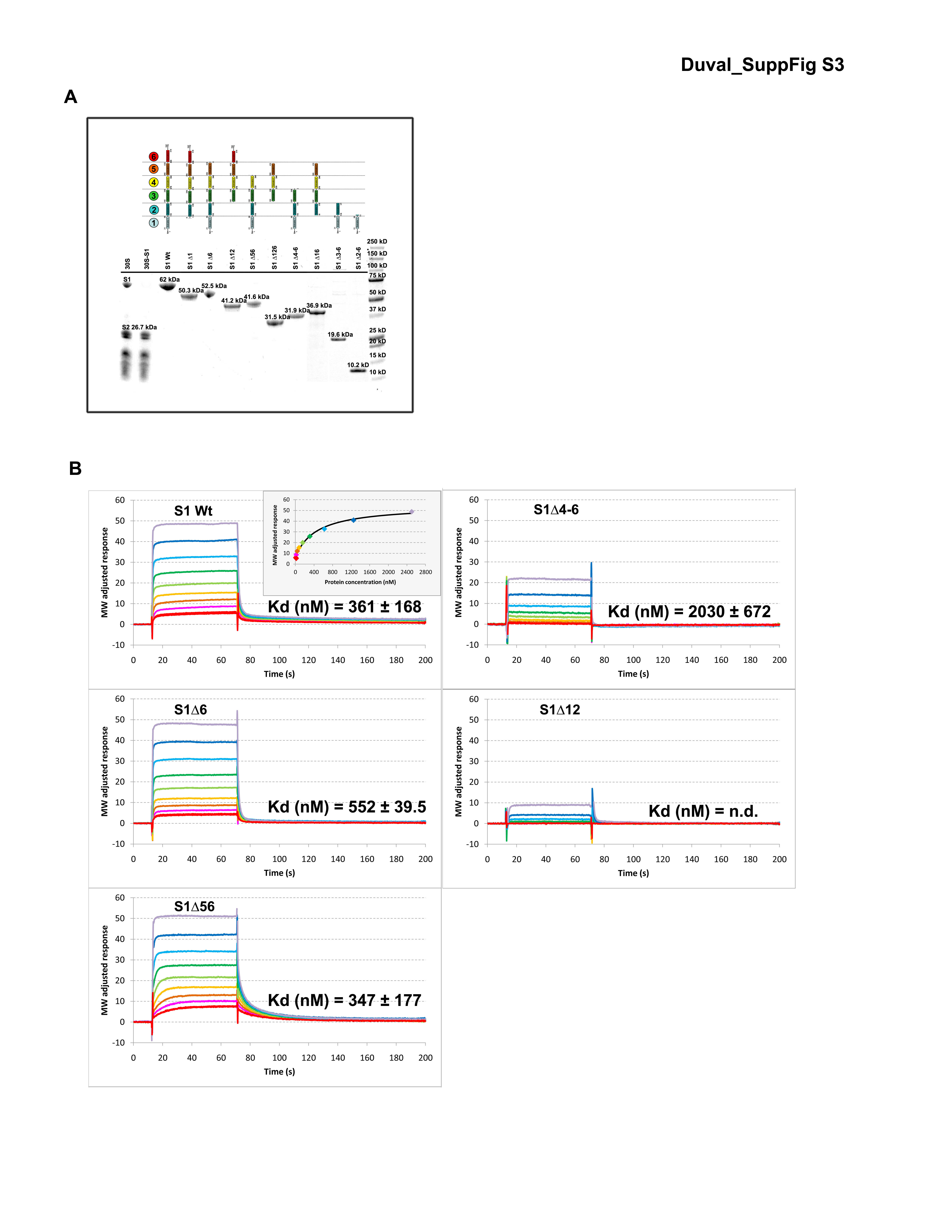

Supplement: Figure S3 — The r-protein S1 mutants and effect of the mutations on RNA binding. (A) Schematic representation of the deletion performed in rpsA for in vitro studies. On the top of the gel, the six OB-fold domains of S1 are represented with different colors. Polyacrylamide-SDS gel electrophoresis was performed on 30S, 30S−S1 (lacking S1), and r-protein S1. Lane 1, 30S; lane 2, 30S−S1 lacking S1; lane 3, wild-type S1 (S1 Wt); lane 4, deletion of domain 1 (S1Δ1); lane 5, deletion of domain 6 (S1Δ6); lane 6, deletion of domains 1 and 2 (S1Δ12); lane 7, deletion of domains 5 and 6 (S1Δ56); lane 8, deletion of domains 1, 2, and 6 (S1Δ126); lane 9, deletion of domains 4 to 6 (S1Δ4–6); lane 10, S1 deletion of domains 1 and 6 (S1Δ16); lane 11, deletion of domains 3 to 6 (S1Δ3–6); lane 12, deletion of domains 2 to 6 (S1Δ2–6). A ladder with various size markers is given. All proteins were purified to homogeneity. (B) SPR real-time sensorgrams showing dose-dependent interaction between psk-rpsOSD mRNA and various proteins. Increasing concentrations of proteins (9 nM in red, 19 nM in pink, 39 nM in orange, 78 nM in yellow, 156 nM in light green, 312 nM in dark green, 625 nM in light blue, 1,250 nM in dark blue, and 2,500 nM in purple) have been injected to the immobilized pseudoknot psk-rpsOSD mRNA (190 RU). As proteins, we used wild-type S1 (S1-WT) or S1 deleted of domain 6 (S1Δ6), of domains 5 and 6 (S1Δ56), of domains 4 to 6 (S1Δ4–6), and of domains 1 and 2 (S1Δ12). Binding curves were double-reference subtracted from buffer blank and reference flow cell (without RNA) and adjusted to the molecular weight of the proteins (Response = (RU/MW)×10,000). SPR was used to determine the KD for psk-S1WT interaction by equilibrium binding measurements. The light grey insert in the top panel is a representative SPR response at equilibrium from three experiments. (JPG) [file pbio.1001731.s003.jpg]

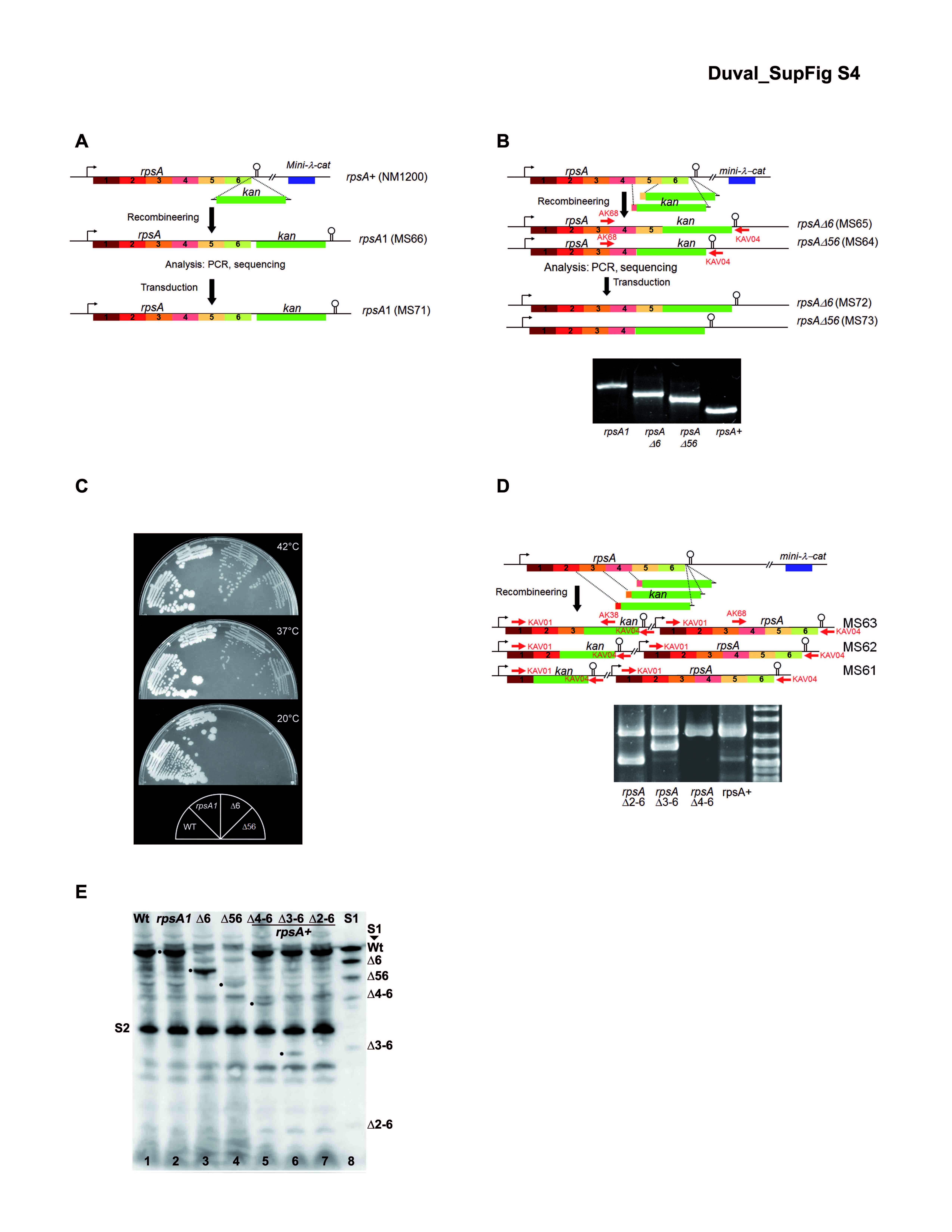

Supplement: Figure S4 — Constructions of rpsA mutant strains in vivo . (A) Construction of the rpsA1 allele. The rpsA gene (with its six domains) is shown with its proximal promoter (rightwards arrow) and its putative terminator (schematised as a stem-loop structure). The drawing is not to scale. A 926 bp long PCR DNA fragment was used to insert kan sequences immediately downstream of the translation termination site of rpsA (see Text S1 for the recombineering protocols). (B) Schematic representation of the construction of the two viable rpsA alleles deleted of domain 6 or domains 5 and 6. The constructs were verified on an agarose gel analysis of the PCR fragments made with the resulting strains (MS65 for Δ6 and MS64 for Δ56) in comparison to rpsA1 (MS66) and wild-type (MG1655). The PCR reaction was performed with oligonucleotides AK68 (complementary to the junction between the domains 3 and 4) and KAV04 (complementary to sequences downstream of rpsA) in the sense and antisense directions, respectively (Table S2). (C) Phenotypic analysis of rpsA alleles deleted of domain 6 or domains 5 and 6. Strains MS78 and MS79 carrying the Δ6 and Δ56 rpsA alleles, respectively, and control strains carrying the WT (AnK02) and rpsA1 (MS77) alleles were streaked on LB plates at the indicated temperatures. (D) Construction of diploid strains. Schematic representation of the constructions and verification of the constructs on an agarose gel analysis of the PCR fragments made with the resulting strains (MS63 for Δ4–6, MS62 for Δ3–6, and MS61 for Δ2–6) in comparison to WT rpsA (MG1655, rpsA+). The PCR reaction was performed with oligonucleotides KAV01 (complementary to sequences in domain 1) and KAV04 in the sense and antisense directions, respectively. (E) Measurement of the levels of the different S1 derivatives in haploid (Δ6 and Δ56) and diploid strains (Δ4–6, Δ3–6, Δ2–6). Western blot of extracts from strains carrying different alleles of rpsA (lanes 1 to 7) was performed using rabbit anti-S1 and anti-S [file pbio.1001731.s004.jpg]

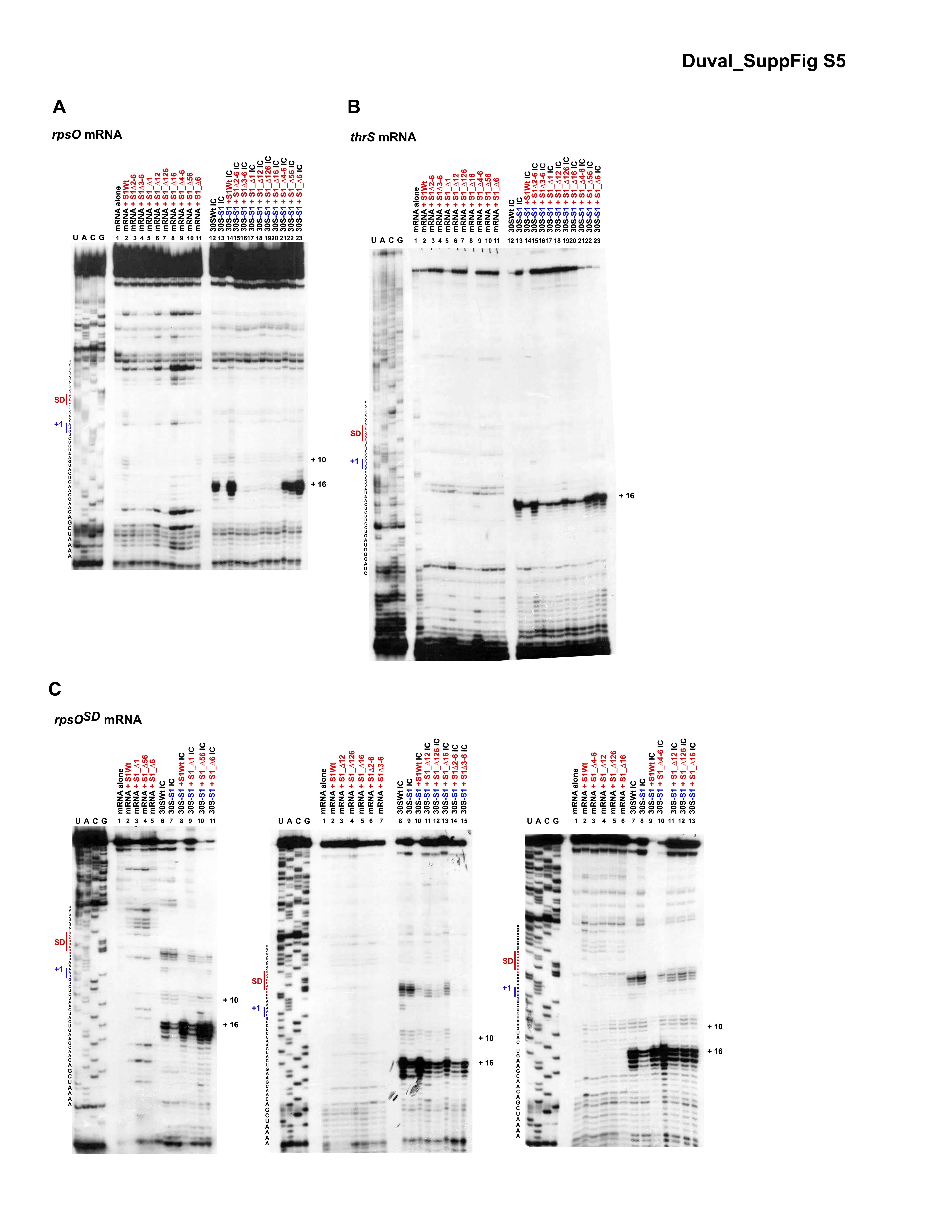

Supplement: Figure S5 — Analysis of the formation of the 30S initiation complex (30SIC) by toeprinting assays. (A) Toeprinting assays performed with wild-type rpsO mRNA (WT rpsO). Lane 1, incubation control of mRNA alone; lanes 2 to 11, incubation controls of mRNA in the presence of WT and the different truncated variants of r-protein S1 as indicated; lane 12, formation of 30SIC formed with WT rpsO, wild-type 30S (30SWt), and initiator tRNAMet; lane 13, formation of 30SIC formed with the 30S lacking S1 (30S−S1); lanes 14–23, 30SIC performed with WT rpsO, tRNA, and 30S−S1 reconstituted with WT S1 (lane 14, 30S+S1), or with S1 deleted of domains 2 to 6 (lane 15, S1Δ2–6), deleted of domains 3 to 6 (lane 16, S1Δ3–6), deleted of domain 1 (lane 17, S1Δ1), deleted of domains 1 and 2 (lane 18, S1Δ12), deleted of domains 1, 2, and 6 (lane 19, S1Δ126), deleted of domains 1 and 6 (lane 20, S1Δ16), deleted of domains 4, 5, and 6 (lane 21, S1Δ4–6), deleted of domains 5 and 6 (lane 22, S1Δ56), or deleted of domain 6 (lane 23, S1Δ6). Lanes U, A, C, and G, sequencing ladders. (B) Formation of the 30SIC involving thrS mRNA, 30S, and the initiator tRNA. Same legend as in the panel A. (C) Formation of the 30SIC involving rpsOSD mRNA, 30S, and the initiator tRNA. The mRNA carries a reinforced SD sequence. Left gel, lane 1, incubation control of mRNA alone; lanes 2 to 5, incubation controls of mRNA in the presence of WT and different truncated r-proteins S1 as indicated; lane 6, formation of the 30SIC formed with WT rpsO, 30SWt, and initiator tRNAMet; lane 7, 30SIC formed with 30S−S1; lanes 8–10, 30SIC formed with WT rpsO, the initiator tRNA, and 30S–S1 reconstituted with S1Δ1 (lane 8), S1Δ56 (lane 9), and S1Δ6 (lane 10). Middle gel, lane 1, incubation control of mRNA alone; lanes 3 to 7, incubation controls of mRNA in the presence of WT and different truncated r-proteins S1 as indicated; lane 8, 30SIC formed with WT rpsO, 30SWt, and initiator tRNAMet; lane 9, 30SIC formed with 30S−S1; lanes 10–14, 30SIC forme [file pbio.1001731.s005.jpg]
