# Supplementary material for: Escherichia coli Ribosomal Protein S1 Unfolds Structured mRNAs Onto the Ribosome for Active Translation Initiation
Source: PLoS Biol. 2013 Dec 10;11(12):e1001731. doi: 10.1371/journal.pbio.1001731 (PMC3858243; doi:10.1371/journal.pbio.1001731)
Supplement: Table S1 — Strains and plasmids used in this study. (DOCX) [file pbio.1001731.s006.docx]

**Supplementary table S1: Strains and plasmids used in this study.**

| **Name of strains/plasmids** | **Genotype** | **Origin/reference** |
| --- | --- | --- |
| AnK02 | *thi-1, argE3, lacI’::bla-rpsA-lacZ, mtl-1, xyl-5, tsx-29?, rpsL, argG6, his-4* | AmpR transductant (acceptor: IBPC5321, donor: MS46) |
| IBPC5321 | *thi-1, argE3, Δlac)X74, mtl-1, xyl-5, tsx-29?, rpsL, argG6, his-4* | [[9](#_ENREF_9)] |
| MG1655 | *rph-1* | Wild type *E. coli* |
| MS02 | *gal490, λdcI857 Δ(cro-bioA), lacI’::bla-cat-sacB* | Replacement of kan by bla. PCR fragment made with oligos AK01 and AK02 using pBR322 as a template recombineered into NC397 |
| MS46 | *gal490, λdcI857 Δ(cro-bioA), lacI’::bla-rpsA-lacZ* | Construction of the rpsA-lacZ fusion. PCR fragment made with oligos AK48 and AK49 using pSP261 as a template recombineered into MS02. |
| MS61 | *rph-1, rpsAΔ2-6::kan/ rpsA+* | PCR fragment made with oligos AK59 and AK61 using pDJ13kan as a template recombineered into NM1200. Cured of mini-λ-cat. |
| MS62 | *rph-1, rpsAΔ3-6::kan/ rpsA+* | PCR fragment made with oligos AK59 and AK62 using pDJ13kan as a template recombineered into NM1200. Cured of mini-λ-cat. |
| MS63 | *rph-1, rpsAΔ4-6::kan/ rpsA+* | PCR fragment made with oligos AK59 and AK63 using pDJ13kan as a template recombineered into NM1200. Cured of mini-λ-cat. |
| MS64 | *rph-1, rpsAΔ56::kan* | PCR fragment made with oligos AK59 and AK64 using pDJ13kan as a template recombineered into NM1200. Cured of mini-λ-cat. |
| MS65 | *rph-1, rpsAΔ6::kan* | PCR fragment made with oligos AK59 and AK65 using pDJ13kan as a template recombineered into NM1200. Cured of mini-λ-cat. |
| MS66 | *rph-1, rpsA1::kan* | PCR fragment made with oligos AK59 and AK66 using pDJ13kan as a template recombineered into NM1200. Cured of mini-λ-cat. |
| MS71 | *rph-1, rpsA1::kan* | KanR transductant (acceptor: MG1655, donor: MS66) |
| MS72 | *rph-1, rpsAΔ6::kan* | KanR transductant (acceptor: MG1655, donor: MS65). |
| MS73 | *rph-1, rpsAΔ56::kan* | KanR transductant (acceptor: MG1655, donor: MS64) |
| MS77 | *thi-1, argE3, lacI’::bla-rpsA-lacZ, rpsA1::kan, mtl-1, xyl-5, tsx-29?, rpsL, argG6, his-4* | KanR transductant (acceptor: AnK02, donor: MS66) |
| MS78 | *thi-1, argE3, lacI’::bla-rpsA-lacZ, rpsAΔ6::kan, mtl-1, xyl-5, tsx-29?, rpsL, argG6, his-4* | KanR transductant (acceptor: AnK02, donor: MS65) |
| MS79 | *thi-1, argE3, lacI’::bla-rpsA-lacZ, rpsAΔ56::kan, mtl-1, xyl-5, tsx-29?, rpsL, argG6, his-4* | KanR transductant (acceptor: AnK02, donor: MS65) |
| MS82pNK34 | *thi-1, argE3, lacI’::bla-rpsA-lacZ, rpsAΔ2-6::kan, mtl-1, xyl-5, tsx-29?, rpsL, argG6, his-4 pNK34* | KanR transductant (acceptor: AnK02 transformed with pNK34, donor: MS61) |
| MS83pNK34 | *thi-1, argE3, lacI’::bla-rpsA-lacZ, rpsAΔ3-6::kan, mtl-1, xyl-5, tsx-29?, rpsL, argG6, his-4 pNK34* | KanR transductant (acceptor: AnK02 transformed with pNK34, donor: MS62) |
| MS84pNK34 | *thi-1, argE3, lacI’::bla-rpsA-lacZ, rpsAΔ4-6::kan, mtl-1, xyl-5, tsx-29?, rpsL, argG6, his-4 pNK34* | KanR transductant (acceptor: AnK02 transformed with pNK34, donor: MS63) |
| MS88 | *thi-1, argE3, Δ(lac)X74, mtl-1, xyl-5, tsx-29?, rpsL, argG6, his-4, mini-λ-cat* | CamR transductant (acceptor: IBPC5321, donor: NM1200) |
| MS97 | *thi-1, argE3, Δ(lac)X74, mtl-1, xyl-5, tsx-29?, rpsL, argG6, his-4, rpsAΔ56::kan* | PCR fragment made with oligos AK59 and AK64 using pDJ13kan as a template recombineered into MS88. Cured of mini-λ-cat. |
| MS98pNK39a | *thi-1, argE3, Δ(lac)X74, mtl-1, xyl-5, tsx-29?, rpsL, argG6, his-4, rpsAΔ4-6::kan pNK34* | PCR fragment made with oligos AK59 and AK63 using pDJ13kan as a template recombineered into MS88 transformed with pNK34. Cured of mini-λ-cat |
| NC397 | *gal490, λdcI857 Δ(cro-bioA), lacI’::kan-cat-sacB* | [[2](#_ENREF_2)] |
| NM1200 | *rph-1, mini-λ-cat* | N. Majdalani (NIH) |
| BL21 (DE) pLysS | *F- ompT gal dcm lon hsdSB(rB*  *-mB-) λ(DE3) pLysS(cmR)* | Invitrogen |
| pBR322 | *tet, bla* | [[10](#_ENREF_10)] |
| pDJ13kan (Tn5) | *kan, bla* | HindIII fragment of Tn5 carrying kan cloned into the HindIII site of BlueScribe M13+ (Cold Spring Harbor Advanced Bacterial Genetics course 1986) |
| pNK34 | *tet; lacIq, Ptrc-rpsA/His6* | BsrG1 fragment of pDErpsA carrying rpsA/His6 cloned into the Acc65I site of pTet99avr |
| pNK39a | *tet; lacIq, sacB, Ptrc-rpsA/His6* | Gene sacB was PCR-amplified with oligos AK89 and AK90 using pSG335 as a template, digested with AseI and cloned into NdeI site of pNK34 |
| pSG335 | *cat, sacB* | [[11](#_ENREF_11)] |
| pSP261 | *cat; rpsA* | [[12](#_ENREF_12)] |
| pTet99a | *tet ; lacIq* | [[13](#_ENREF_13)] |
| pTet99avr | *tet; lacIq* | Replacement of NcoI site by AvrII in the pTet99a by PCR using oligos AK79 and AK80 |
| pETrpsA | *rpsA* wt with C-ter His tag*,* AmpR | [[14](#_ENREF_14)] |
| pETrpsA_R227C | *rpsA* mutated (C679T, +1 being the A of start AUG) *bla* | Obtained by mutagenesis of the pETrpsA plasmid using the DpnI system with the QuickChange kit (Qiagen) using the appropriate oligonucleotides (Table S2). |
| pETrpsA∆6 | *rpsA*∆*6* (aa1 to aa465 with C-ter His tag), *bla* | PCR fragment containing the mutation was done with the appropriate oligos on the pETrpsA template (see Table S2) and cloned into pETrpsA plasmid between the NdeI – XhoI sites. |
| pETrpsA∆12 | *rpsA*∆12 (aa191 to aa557 with C-ter His tag), *bla* | PCR fragment containing the mutation was done with the appropriate oligos on the pETrpsA template (see Table S2) and cloned into pETrpsA plasmid between the NdeI – KpnI sites. |
| pETrpsA∆126 | *rpsA*∆126 (aa191 to aa465 plus Cter His tag), *bla* | PCR fragment containing the mutation was done with the appropriate oligos on the pETrpsA template (see Table S2) and cloned into pETrpsA plasmid between the NdeI – XhoI sites. |
| pUC18_psk | *rpsO-psk*, *bla* | Cloning into the pUC18 plasmid (Invitrogen) the DNA fragment obtained by annealing 6 oligonucleotides (see Table S2) between the EcoRI – BamHI sites. |
| pUC18_mut-psk | *rpsO-mut-psk*, *bla* | Obtained by mutagenesis of the pUC18_psk plasmid using the DpnI system with the QuickChange kit (Qiagen) and appropriate oligonucleotides (Table S2). |
| pDErpsA | *bla rpsA*/*His_6_* | Ligation of the *BglI*I-*Mlu*I fragment from pDEST_Δ4-6 to the *BglI*I-*Mlu*I fragment of pDEST_Δ1. |
| pDEST14 | *bla* | Invitrogen |
| pDEST_∆4-6 (4207) | *rpsA*∆*4-6* (aa1 to aa279 with C-ter His tag), *bla* | Obtained with the Gateway Cloning Strategy (Life technologies, Invitrogen). The list of oligos used can be provided upon request. |
| pDEST_∆56 (4209) | *rpsA∆56* (aa1 to aa365 with C-ter His tag), *bla* | Obtained with the Gateway Cloning Strategy (Life technologies, Invitrogen). The list of oligos used can be provided upon request. |
| pDEST_∆1 (4210) | *rpsA∆1* (aa110 to aa557 with C-ter His tag), *bla* | Obtained with the Gateway Cloning Strategy (Life technologies, Invitrogen). The list of oligos used can be provided upon request. |
| pDEST_∆16 (4211) | *rpsA∆16* (aa110 to aa432 with C-ter His tag), *bla* | Obtained with the Gateway Cloning Strategy (Life technologies, Invitrogen). The list of oligos used can be provided upon request. |
| pDEST_∆2-6 (4201) | *rpsA∆2-6* (aa1 to aa86 with C-ter His tag), *bla* | Obtained with the Gateway Cloning Strategy (Life technologies, Invitrogen). The list of oligos used can be provided upon request. |
| pDEST_∆3-6 (4204) | *rpsA*∆3-6 (aa1 to aa170 with C-ter His tag), *bla* | Obtained with the Gateway Cloning Strategy (Life technologies, Invitrogen). The list of oligos used can be provided upon request. |
