# Supplementary material for: Escherichia coli Ribosomal Protein S1 Unfolds Structured mRNAs Onto the Ribosome for Active Translation Initiation
Source: PLoS Biol. 2013 Dec 10;11(12):e1001731. doi: 10.1371/journal.pbio.1001731 (PMC3858243; doi:10.1371/journal.pbio.1001731)
Supplement: Table S2 — List of oligonucleotides. (DOCX) [file pbio.1001731.s007.docx]

**Supplementary table S2: List of oligonucleotides**

| **Name** | **Usage** | **Sequences are 5’-end to 3’end** |
| --- | --- | --- |
| AK01 | Replacement *kan* by *bla* in NC397 | ACTGTCTGCTTACATAAACAGTAATACAAGGGGTGTTATGAGTATTCAACATTTCCGTG |
| AK02 | Replacement *kan* by *bla* in NC397 | TGCTCTGCCAGTGTTACAACCAATTAACCAATTCTGATTACCAATGCTTAATCAGTGAG |
| AK07 | The lac fusion check/sequencing | GTGAACGCTCTCCTGAGTAGG |
| AK08b | The lac fusion check/sequencing | CATTCGCCATTCAGGCTGCGCAAC |
| AK38 | PCR analysis of *rpsA* replacements | GCCATGATGGATACTTTCTCGGC |
| AK48 | *rpsA-lacZ* construction, forward | GAATTAAAATGAGACGTTGATCGGCACGTAAGAGGTTCCACCACCTTAAGCATTGAGC |
| AK49 | *rpsA-lacZ* construction, reverse | AGTCACGACGTTGTAAAACGACGGCCAGTGAATCCGTAATGCGGGTTTCGATTTCTTTTAAGG |
| AK59 | All *rpsA* replacements, reverse | CAACAAACTTCGGAATAAAAATCCCGAAGAGTCAGAGAATTAGAAGAACTCGTCAAGAAGGCG |
| AK61 | *rpsAΔ23456* construction, forward | CGCAGTAGAAGACGGCTTCGGTGAAACTCTGCTGTCCCGTTAATGGCGCAGGGGATCAAGATC |
| AK62 | *rpsAΔ3456* construction, forward | GCTGGATCAGAAGCGCAACAACGTTGTTGTTTCTCGTCGTTAATGGCGCAGGGGATCAAGATC |
| AK63 | *rpsAΔ456* construction, forward | GTGGGTAGCTATCGCTAAACGTTATCCGGAAGGTACCAAATAATGGCGCAGGGGATCAAGATC |
| AK64 | *rpsAΔ56* construction, forward | CCCGTGGCAGCAGTTCGCGGAAACCCACAACAAGGGCGACTAATGGCGCAGGGGATCAAGATC |
| AK65 | *rpsAΔ6* construction, forward | TCTGCAGGTTGACGCAGAACGTGAACGTATCTCCCTGGGCTAATGGCGCAGGGGATCAAGATC |
| AK66 | *rpsA1* construction, forward | ACGCAATGGCTGAAGCTTTCAAAGCAGCTAAAGGCGAGTAATGGCGCAGGGGATCAAGATC |
| AK68 | PCR analysis of *rpsA* replacements | CCGGAAGGTACCAAACTGACTG |
| AK79 | pTet99avr construction | GGAAACAGACCTAGGAATTCGAGC |
| AK80 | pTet99avr construction | GCTCGAATTCCTAGGTCTGTTTCC |
| AK89 | pNK39a construction | GGCAAATTAATGCCCATGCAACAG |
| AK90 | pNK39a construction | GCTCTGGGAGGCAGAATTAATGATCC |
| KAV01 | PCR analysis of *rpsA* replacements | GCTGGAAATCCAGGTAGGTG |
| KAV04 | PCR analysis of *rpsA* replacements | GCATTGATTAAGGGCGGCCGTAG |
| T7_*sodB* | PCR amplification of *sodB* gene | GGATCCTAATACGACTCACTATAGGATACG CACAATAAGGC |
| *sodB*_Rev | PCR amplification of *sodB* gene | CCGCAGAAATGTGCGGTG |
| *sodB*_Rev2 | Toeprinting assay on *sodB* | GAAATGTGCGGTGCCAG |
| 38-50 | Toeprint *rpsO* | CGACCAAACTCAG |
| *thrS* toeprint | Toeprint *thrS* | CTTGGGCTTACAGCG |
| R227C | Mutagenesis for SDCP | CACTGACATGGCCTGGAAATGCGTTAAGCATCC |
| R227C_anti | Mutagenesis for SDCP | GGATGCTTAACGCATTTCCAGGCCATGTCAGTG |
| psk_1 | To produce psk fragment of *rpsO* mRNA | AATTCAATACGACTCACTATAGGGATC |
| psk_2 | To produce psk fragment of *rpsO* mRNA | GCTGAATTAGAGATCGGCGTCCTTTCATTCTA |
| psk_3 | To produce psk fragment of *rpsO* mRNA | TATACTAAGGAGGTTAAAATGTCTCTAAGTACTG |
| psk_4 | To produce psk fragment of *rpsO* mRNA | GATCTCTAATTCAGCGATCCCTATAGTGAGTCGTATTG |
| psk_5 | To produce psk fragment of *rpsO* mRNA | ACCTCCTTAGTATATAGAATGAAAGGACGCC |
| psk_6 | To produce psk fragment of *rpsO* mRNA | GATCCAGTACTTAGAGACATTTTA |
| mut-psk | To produce mut-psk fragment of *rpsO* mRNA | GAGATCGGCGTCCTTTCATTCTATATAGTAAGGAGGTTAAAAT |
| mut-psk_anti | To produce mut-psk fragment of *rpsO* mRNA | ATTTTAACCTCCTTACTATATAGAATGAAAGGACGCCGATCTC |
| *rpsA∆6* | S1 ∆6 plasmid construction | TAACTTTAAGAAGGAGATATACATATGACTGAATCTTTTGCTCAAC |
| *rpsA∆6*_anti | S1 ∆6 plasmid construction | GTGGTGGTGGTGCTCGAGGCCTTTAGCGTCAACTG |
| *rpsA∆12* | S1 ∆12 plasmid construction | CATATGGGCATGGAAGTTAAAGGTATG |
| *rpsA∆12*_anti | S1 ∆12 plasmid construction | CCAGTCAGTTTGGTACCTTCCG |
| *rpsA∆126* | S1 ∆126 plasmid construction | CATATGGGCATGGAAGTTAAAGGTATG |
| *rpsA∆126*_anti | S1 ∆126 plasmid construction | CCAGTCAGTTTGGTACCTTCCG |
