# Supplementary material for: Escherichia coli Ribosomal Protein S1 Unfolds Structured mRNAs Onto the Ribosome for Active Translation Initiation
Source: PLoS Biol. 2013 Dec 10;11(12):e1001731. doi: 10.1371/journal.pbio.1001731 (PMC3858243; doi:10.1371/journal.pbio.1001731)
Supplement: Text S1 — Supplementary experimental procedures. (DOCX) [file pbio.1001731.s008.docx]

**Supplementary information**

***Escherichia coli* ribosomal protein S1 unfolds structured mRNAs onto the ribosome for active translation initiation**

Mélodie Duval^1^, Alexey Korepanov^2*^, Olivier Fuchsbauer^1^, Pierre Fechter^1^, Andrea Haller^3^, Attilio Fabbretti^4^, Laurence Choulier^5^, Ronald Micura^3^, Bruno Klaholz^6^, Pascale Romby^1§^, Mathias Springer^2^ and Stefano Marzi^1§^

^1^Architecture et Réactivité de l’ARN, Université de Strasbourg, IBMC-CNRS, F-67084 Strasbourg, France; ^2^CNRS UPR9073, University Paris Diderot, Sorbonne Paris Cité, institut de Biologie Physico-Chimique, 75005 Paris, France; ^3^Institute of Protein Research, Russian Academy of Sciences, 4 Institutskaya str., Pushchino, Russia 142290; ^4^Institute of Organic Chemistry and Center for Molecular Biosciences, Leopold Franzens University, Innrain 80-82, A-6020 Innsbruck, Austria; ^5^Laboratory of Genetics, Dept. of Biology MCA, University of Camerino, 62032 Camerino, Italy; ^6^CNRS UMR 7213, Université de Strasbourg, Faculté de Pharmacie 67401 Illkirch, France; ^7^Department of Integrated Structural Biology, IGBMC, Illkirch, France.

**Supplementary Experimental Procedures**

**Plasmids and strain constructions**

The plasmid pTet99avr was obtained from pTet99a by replacing the *Nco*I site by an *Avr*II site using PCR-amplification with oligos AK79 and AK80 according to the QuikChange Site-Directed Mutagenesis Kit protocol (Stratagene, USA) using KOD Hot start DNA-polymerase (Novagen, Japan). Plasmid pNK34 was constructed by cloning the *Bsr*G *rpsA/His_6_*-containing fragment of pDESTrpsA into the *Acc*65I site of pTet99avr. The plasmid pDErpsA (a generous gift from Anna Korobeinikova) was constructed by ligating the *BglI*I-*Mlu*I fragment from pDEST_Δ4-6, which carries the start of the *rpsA* gene to the *BglI*I-*Mlu*I fragment of pDEST_Δ1, which carries the terminal end of the gene, reconstituting a whole *rpsA*/*His_6_* gene. Thus, *rpsA/His_6_* is expressed from IPTG-inducible hybrid *trc* promoter. To construct pNK39a, the *sacB* gene has been PCR-amplified with oligos AK89 and AK90, using pSG335 as a template. The PCR fragment was digested with *Ase*I and cloned into *Nde*I site of the pNK34. In the resulting plasmid, *sacB* has same orientation as *tet*.

Strains MS02, MS46 and different *rpsA* alleles were constructed using recombineering techniques as previously described in detail [[1](#_ENREF_1)]. Briefly, the selected target sequences of chromosomal DNA were replaced with high efficiency with the PCR cassettes bearing a selectable marker and short (~40 bp) extensions at the 5’- and 3’-termini. These extensions are identical to chromosomal sequences surrounding the sequence to be replaced, thus allowing precise replacement *via* homologous recombination provided by recombinogenic functions of phage λ. To make MS02, the *bla* ORF of pBR322 was PCR-amplified using oligos AK01 and AK02. The obtained PCR cassette was used to precisely replace *kan* ORF by *bla* ORF in the NC397 strain, selecting on LB-ampicillin plates. The NC397 strain [[2](#_ENREF_2)] carries the construction *lacI’::kan-cat-sacB-lacZ* at chromosomal *lac* locus. For the construction of MS46, we recombined into MS02 the PCR fragment containing the original promoter *rpsAp3*, the 5’-UTR and the 19 first codons of *rpsA* made with oligos AK48 and AK49 using pSP261 as a template. The PCR cassette was designed to replace *cat-sacB* cassette and (in frame) the first 3 codons of *lacZ*, thus making an *rpsA-lacZ* translational fusion. The recombinants were selected on LB agar without sodium chloride supplemented with 7% sucrose. Sucrose-resistant clones were verified for sensitivity to chloramphenicol and by colony PCR using oligos AK07 and AK08b that are complementary to the chromosome regions surrounding the targeted locus.

To obtain chromosomal *rpsA* alleles, expressing S1 proteins truncated for different C-terminal domains, we made series of successive deletions of the 3’-proximal sequences of *rpsA* gene by replacing them with the selectable *kan* marker. Several 928 bp long *kan* PCR cassettes were made with forward oligos AK61, AK62, AK63, AK64 and AK65 using pDJ13kan as a template; in all the cases AK59 was used as the reverse oligonucleotide. The PCR cassettes were recombined into NM1200. Recombinants grew on LB-agar supplemented with kanamycin at a concentration of 30 µg/ml within 1-6 days, giving rise to strains MS61, MS62, MS63, MS64 and MS65, respectively. NM1200 carries a curable (at high temperature) mini-λ conferring CamR and allowing the expression of the phage recombination genes at high temperature [[3](#_ENREF_3)]. The cassettes were made to insert a nonsense codon immediately downstream of domain 1 at codon 87 (*rpsAΔ2-6::kan*, MS61), domain 2 at codon 172 (*rpsAΔ3-6::kan*, MS62), domain 3 at codon 280 (*rpsAΔ4-6::kan*, MS63), domain 4 at codon 366 (*rpsAΔ56::kan* MS64) or domain 5 at codon 433 (*rpsAΔ6::kan*, MS65). The *kan* ORF always begins 51 bp downstream of the inserted nonsense codon, which is followed by the sequences situated downstream of the original WT *rpsA* ORF. Thus, *kan* is transcriptionally fused to *rpsA* but is expressed from its own SD sequence. As a control, similar 926 bp long PCR cassette was made with oligos AK66 and AK59 to insert the *kan* cassette downstream of wild type *rpsA* ORF (*rpsA1::kan*, strain MS66), providing a strain synthesizing a full-size S1 protein. The *kan* insertions were verified by colony PCR with oligonucleotides KAV01 and KAV04. As the PCR fragment for *rpsAΔ4-6::kan* has the size of that of WT *rpsA*, the presence of the WT allele was verified with the oligonucleotides AK68 (complementary to the junction between domains 3 and 4) and KAV04. The presence of mutant alleles was checked with the oligonucleotides KAV01 and AK38 (reverse oligonucleotide, complementary to sequences within the *kan* cassette)

The routine generalized transduction by phage P1 was used for allele transfer [[4](#_ENREF_4)]. The *kan* marker was used to transduce viable *rpsA* alleles to MG1655 to yield strains MS71 (*rpsA1*), MS72 (*rpsAΔ6*) and MS73 (*rpsAΔ56*) or to AnK02 in order to obtain strains MS77 (*rpsA1*), MS78 (*rpsAΔ6*) and MS79 (*rpsAΔ56*). Growth characteristics of these strains were estimated by growth on LB plates at 37⁰C and liquid LB medium at the same temperature (Figures 4A-B). Doubling time was calculated by plotting the culture optical density at 600 nm versus time in semi-logarithmic graph (not shown).

The larger deletions *rpsAΔ2-6, rpsAΔ3-6* and *rpsAΔ4-6* were obtained as haploids by transduction from diploid strains MS62, MS63 and MS64, respectively, to strain AnK02 transformed with the complementing plasmid pNK34. Transductants were selected on LB plates supplemented with 30 µg/ml kanamycin, 12.5 µg/ml tetracycline and 3-5 µM IPTG, providing strains MS82pNK34, MS83pNK34 and MS84pNK34, respectively. The 3-5 µM IPTG concentration allows the synthesis of S1 levels adequate for complementation with pNK34, while higher IPTG concentrations are toxic due to S1 overproduction. To see whether these strains are viable in the absence of IPTG, they were grown on the LB plates supplemented with kanamycin, tetracycline in the presence or in the absence of IPTG (Figure 4C) at 37⁰C. The viable MS79 strain transformed with pNK34 was used as a control. To estimate growth of viable *rpsA* alleles at different temperatures, strains AnK02 (WT S1), MS77 (*rpsA1*), MS78 (Δ6) and MS79 (Δ56) were grown on LB-agar plates at 42⁰C, 37⁰C or 20⁰C (Figure S4C).

We also tried to cure strains carrying the *rpsAΔ4-6* allele from the complementing plasmid. To achieve this, we constructed the strain MS98pNK39a by recombineering *rpsAΔ4-6::kan* into MS88 transformed with the plasmid pNK39a. The recombinants were selected on LB plates supplemented with kanamycin, tetracycline and 50 µM IPTG. Plasmid pNK39a is a pNK34 derivative that contains *sacB* gene, which is lethal on sucrose-containing media allowing a first counter-selection against cells carrying pNK39a. In the presence of 1 mM IPTG, this plasmid overproduces S1, which is also lethal, allowing a second counter-selection. Several independent clones of MS98pNK39a were purified and curing trials were performed by growing the strain for 40 h in liquid LB medium without any selection and 10^5^ to 10^6^ viable cells plated on the LB plates without sodium chloride, supplemented with 7% sucrose and 1 mM IPTG. The strain MS97 (carrying *rpsAΔ56::kan*, a viable allele) transformed with pNK39a cells was used as a control. Plates were incubated for 8 days. Under our conditions, MS98pNK39a did not loose the plasmid since all plated cells were killed by the excess of IPTG (due to S1 overproduction) and by the presence of sucrose.

The construction of the pDEST plasmids (Table S1) expressing the truncated forms of r-protein S1 for protein purification was done with the Gateway Cloning Strategy (Life technologies, Invitrogen). The list of oligonucleotides used can be provided upon request. The construction of the pETrpsA∆6, pETrpsA∆12 and pETrpsA∆126 has been done using the template pETrpsA plasmid by PCR amplification with the appropriate oligonucleotides (Table S2) and subcloning into the original pETrpsA plasmid using the restriction sites specified in Table S1. The mutation R227C was introduced in *rpsA* gene using the *Dpn*I system with the QuickChange kit (Qiagen) and the appropriate oligonucleotides (Table S2).

***In vivo* quantification of wild type and variant forms of protein S1**

To quantify the content of S1 and its truncated versions, western blotting was used. Usually, 1 OD unit of cells were suspended in 50 μl of SDS-sample buffer, boiled and 6 μl of the assays were loaded on the NuPAGE 12% Bis-Tris precast gel. Gel was run in MES-SDS buffer in Novex Mini-Cell electrophoresis unit (Invitrogen, USA). Proteins were transferred to the PVDF Hybond-P membrane pore size 0.45 μm (Amersham) using XCell II Blot Module (Invitrogen, USA).

The membranes were incubated with rabbit anti-S1 and anti-S2 anti serum (generous gifts from Knut Nierhaus and Irina Boni, respectively), then with mouse anti-rabbit IgG-HRP conjugates, visualized using Immune-Star WesternC Kit and Molecular Imager ChemiDoc XRS+ system (Bio-Rad, USA). The images were quantified using ImageLab software by the same manufacturer. Protein S2 band was used as a loading control and all the other bands to quantify were normalized to it. After normalization, WT S1 content was calculated as a ratio of S1 band density in the studied strains (BD_wt_) to the density of S1 band in MG1655 (BD_ref_) multiplied by 100 (to express results in percentage rather than decimals). For calculating the mutant S1 content, the corresponding band density (BD_mut_) was amplified by the mass ratio (MR, ratio of the Mw of WT S1 to the Mw of the mutant form) and the antibody reactivity factor RF (this is the ratio between densities of WT S1 and the mutant form, when both are loaded in equal quantities (15 ng, Figure S4E, lane 8). The value was divided by BD_ref_ and multiplied by 100. The reactivity factor reflects the fact, that different S1 mutants have different reactivities for anti-S1 anti-serum. The obtained values for S1 content represent, thus, molecular ratios, because mass difference was taken into account. The final formula for S1 content calculation: C= (BD_mut_/BD_ref_)*MR*RF*100.

**Purification of WT, mutated and truncated S1 r-proteins**

Overexpression and purification of the proteins carrying 6 histidines at their C-terminus were done using affinity chromatography followed by a monoQ. For each protein, after cell growth and grinding, the soluble fraction of the protein was first centrifuged to pellet the membranes (30 min at 6000 rpm at 4°C). The protein was then dissociated from the ribosomes by the addition of 1,08 g of NH_4_Cl (1 M final) and the ribosomes were removed by ultracentrifugation (50000 rpm, 2 h). The proteins were first purified on Ni-NTA affinity column, dialyzed, and purified on a mono Q column (pH 7.5, using a gradient of NH_4_Cl from 40 mM to 1 M). The proteins were dialyzed and stored at -20°C in the storage buffer (20 mM Tris HCl pH 7.5, 40 mM NH_4_Cl, 60 mM KCl, 1 mM DTT, 10% glycerol). Mass spectrometry and dynamic light scattering were used to verify the quality of the proteins (results not shown). Protein S1 concentration has been determined by their absorbance at 280 nm using ε_280_=47565 M^-1^ cm^-1^ for WT S1, ε_280_=33585 M^-1^ cm^-1^ for S1∆16, ε_280_=6990 M^-1^ cm^-1^ for S1∆3-6, ε_280_=20970 M^-1^ cm^-1^ for S1∆4-6, ε_280_=33460 M^-1^ cm^-1^ for S1∆56, ε_280_=40575 M^-1^ cm^-1^ for S1∆1, ε_280_=46075 M^-1^ cm^-1^ for S1∆6, ε_280_=40575 M^-1^ cm^-1^ for S1∆12, ε_280_=33585 M^-1^ cm^-1^ for S1∆6. Quantification via Bradford assay (Pierce) has been obtained for S1∆2-6 mutant that do not contain any Trp, Tyr or Cys residues. All S1 preparations have been checked by mass spectrometry. We show that all S1 proteins were of high grade of purity and completely devoided of traces of Hfq. This protein is a natural Ni-NTA binder often found as contaminant in preparations of His-tagged protein when only a single affinity purification step is used [[5](#_ENREF_5)]. We have also introduced a factor Xa cleavage site just before the His-tag to be able to remove the tag after the purification. Proteins with or without the His-tag showed very similar activities in RNA binding (EMSA) or in stimulating the 30SIC formation (toeprinting).

**Purification of r-protein S15**

*E. coli* r-protein S15 was purified under non-denaturing conditions as described by [[6](#_ENREF_6)] except that the protein was purified by two additional chromatographies, an hydroxyapatite (Bio-Rad) followed by a Superdex 75 gel filtration (Pharmacia Biotech).

**Preparation of the 30S and of the 30S lacking r-protein S1**

*E. coli* 30S subunits were purified on sucrose gradients after dissociation of the 70S ribosomes according to Fechter et al. [[7](#_ENREF_7)]. Ribosomal protein S1 was dissociated from the 30S and removed using a polyU Sepharose 4B chromatography (GHealthcare) as follows. The 30S (15 µM) were first incubated 10 min at 37°C in the dissociation buffer containing 20 mM Tris-HCl pH 7.5, 10 mM MgCl_2_, 60 mM KCl, 1 M NH_4_Cl, 1 mM DTT. The samples were loaded on a polyU-sepharose 4B column pre-equilibrated with the dissociation buffer to retain r-protein S1. The 30S^-S1^ particles were eluted with 15 ml of the dissociation buffer. The fractions containing the 30S^-S1^ were pooled and dialyzed overnight in the storage buffer containing 20 mM Tris-HCl pH 7.5, 10 mM MgCl_2_, 50 mM KCl, 1 mM DTT, 0.1 mM EDTA. The absence of S1 in the 30S preparation was verified on a 12% polyacrylamide-SDS gel electrophoresis followed by mass spectrometry.

**Toeprinting assays**

This approach is based on the inhibition of reverse transcription due to the formation of the ternary 30S-mRNA-tRNA complex, which results in a pause corresponding to the 3’ edge of the mRNA bound to 30S. When the mRNA occupies the decoding channel, the toeprint occurs at position +16 (+1 being the adenine of the AUG codon). The formation of the simplified translational initiation complex (30SIC) with mRNA was done using published procedure [[7](#_ENREF_7)]. Prior the toeprinting assays, the 30S subunits were first incubated at 37°C for 10 min. In parallel, the mRNA (0.5-1 pmole) was annealed to a 5’ end-labeled oligonucleotide (100000 cpm per reaction) in 20 mM Tris-HCl pH 7.5, 60 mM NH_4_Cl, 1 mM DTT, heated at 90°C for 1 min and cooled on ice for 1 min. MgCl_2_ was added at 10 mM final concentration and incubation was continued at 20°C for 20 min. WT and mutant r-proteins S1, WT 30S, 30S^-S1^ and 30S^+S1^ were incubated 15 min at 37°C in the toeprinting buffer containing 20 mM Tris-HCl pH 7.5, 60 mM NH_4_Cl, 10 mM MgCl_2_ and 1 mM DTT. The 30S^-S1^ subunits were reconstituted by adding purified r-protein S1 at a ratio 1:3 (30S^-S1^:S1). 30SIC was performed at 37°C for 15 min in the toeprinting buffer in the presence of the mRNA annealed to the labeled primer, 0.2-0.5 µM *E coli* 30S and 1 to 5 µM initiator tRNA^fMet^. The ribosomal entrapped *rpsO* mRNA-30S-S15 was formed at 37°C for 15 min in the toeprinting buffer containing 7.5 mM MgCl_2_ in the presence of the *E. coli* 30S (0.4 µM), *rpsO* mRNA (0.6 µM), and when required r-protein S15 (1 µM) [[8](#_ENREF_8)]. *E. coli* r-protein S15 was previously reactivated for 30 min at 37 °C in 50 mM Tris-HCl pH 7.5, 270 mM KCl, 3 mM DTT, 20 mM MgCl_2_ and 0.02 mg/ml BSA. Primer extension reactions were subsequently performed by adding 2-4 units of AMV reverse transcriptase (RT) or 10 units of MMLV RT at 37°C for 15 min. Reactions were stopped by phenol extraction followed by ethanol precipitation, and samples were loaded on 12% PAGE.

**Interaction of the 30S with r-protein S1**

Purified WT and truncated S1 proteins (50 pmoles) were incubated with 30S^-S1^ (50 pmoles) for 15 min at 37°C in 20 µl of a buffer containing 20 mM Tris-HCl pH 7.5, 60 mM KCl, 40 mM NH_4_Cl, 10 mM MgCl_2_, 3 mM DTT, and 0.02 mg/ml BSA. The complex was then purified on a Superdex 200 HR 10/30 (GE healthcare) column equilibrated with the same buffer. The various fractions were collected and analyzed on a SDS-polyacrylamide gel electrophoresis (PAGE). The fractions containing the 30S were precipitated with 3 volumes of ethanol while the fractions containing the unbound r-protein S1 were precipitated with acetone. After precipitation, the 30S and the free r-protein S1 were run on a 4-12% SDS-PAGE. Controls containing defined amount of 30S or of the purified WT and truncated S1 proteins were run in parallel. Gels were transferred to a nitrocellulose membrane and western blots were done using antibodies directed against the His-tag present at the C-terminus of each S1. Each band was then quantified using TotalLab TL100 v2008 software (Nonlinear Dynamics). The amount of bound and unbound proteins was calculated by calibration with the known amount of the purified proteins loaded in the control lanes.

**Formation of 30S-mRNA complexes by filtration on nitrocellulose**

The reactions were performed at 37 °C for 15 min in 60 µl containing 20 pmoles of 30S or 30S^-S1^, 40 pmoles of initiator tRNA_f_^Met^ and various concentrations of 5’ end-labeled mRNA (0,5, 10, 20, 30, and 40 pmoles) in 20 mM Tris pH 7.5, 60 mM NH_4_Cl, 1 mM DTT, and 10 mM MgCl_2_. The mix of cold and radiolabeled mRNA (final activity 5000 cpm/µl) was previously renatured as described above. The mixture was then filtrated on nitrocellulose membrane and the radioactivity was measured. Three independent experiments were performed for each mRNA. The graph presented in Figure S1B referred to the average of three experiments including the error bars obtained using the standard deviations.

**Gel retardation assays**

The end-labeled RNA was denatured at 90°C for 1 min in a buffer containing 20 mM Tris-HCl pH 7.5, 60 mM NH_4_Cl, and 1 mM DTT. After 1 min in ice, renaturation was performed at 20°C for 20 to 30 min in the presence of 10 mM MgCl_2_. Protein S1 was pre-incubated for 15 min at 37°C in the S1 buffer containing 20 mM Tris-HCl pH 7.5, 10 mM MgCl_2_, 60 mM KCl, 40 mM NH_4_Cl, 3 mM DTT, and 0.02 mg/ml BSA. Complex formation was performed at 37°C for 15 min with 5’ end-labeled RNA (12000 cpm) and increasing concentrations of r-protein S1 in 10 µl of S1 buffer. When necessary, RNasin (4 U/µl) was added to the assays. After the addition of 10 µl of glycerol-dyes, the samples were migrated on a 10% PAGE under non-denaturing conditions.

**Surface Plasmon Resonance assays**

All experiments were performed on a Biacore T100 instrument (GE Healthcare) at 25°C using the sCM5 sensor surfaces (BR-1006-68, GE Healthcare). The running buffer was composed of 10 mM Tris-HCl, pH 7.5, 10 mM magnesium acetate, 100 mM ammonium acetate, 0.005% tween20. We added DTT 1 mM to the samples containing proteins S1 or the ribosomes. For RNA-protein binding experiments, biotinylated *rpsO* mRNA fragment containing only the pseudoknot structure were captured on streptavidine (Sigma) previously immobilized in a covalent fashion to carboxyl groups of the dextran matrix using the standard amine coupling protocol (Amine Coupling Kit, BR-1000-50, GE Healthcare). Briefly, sCM5 sensor surfaces were activated for 10 min with a 1:1 mix of 0.2 M N-ethyl-N’-(3-dimethylaminopropyl)-carbodiimide hydrochloride (EDC) and 0.05 M N-hydroxysuccinimide (NHS). Streptavidine was then injected to reach a level of 15000-16000 RU. Remaining activated groups were blocked with 1 M ethanolamine, pH 8.5. Biotinylated RNAs diluted in the running buffer were injected and captured on the flow cells. These procedures resulted in surfaces with 190 RU of mRNA. Blank surface was obtained by treating the surface as for the RNA capture, except that the RNA injection was omitted. Protein samples at 9, 19, 39, 78, 156, 312, 625, 1250, 2500 nM were then injected consecutively for 60s at a flow rate of 30 µl/min. The regeneration step was done by two successive injections of NaCl 1.5 M.

Responses were XY-zeroed and double-referenced (i.e., the sensorgrams were corrected for signals in the reference flow cell, as well as for the blank buffer data). To facilitate data comparison, responses were normalized relative to the molecular weight (MW) of injected proteins. Results were interpreted using the Biacore T100 Evaluation software 2.0.2 (GE Healthcare).

**Supplementary References**

1. Thomason L, Court DL, Bubunenko M, Costantino N, Wilson H, et al. (2007) Recombineering: genetic engineering in bacteria using homologous recombination. Curr Protoc Mol Biol Chapter 1: Unit 1 16.

2. Svenningsen SL, Costantino N, Court DL, Adhya S (2005) On the role of Cro in lambda prophage induction. Proc Natl Acad Sci U S A 102: 4465-4469.

3. Court DL, Swaminathan S, Yu D, Wilson H, Baker T, et al. (2003) Mini-lambda: a tractable system for chromosome and BAC engineering. Gene 315: 63-69.

4. Miller J (1972) Experiments in Molecular Genetics. Cold Spring Harbor, NY: Cold Spring Harbor Laboratory.

5. Milojevic T, Grishkovskaya I, Sonnleitner E, Djinovic-Carugo K, Blasi U (2013) The Pseudomonas aeruginosa Catabolite Repression Control Protein Crc Is Devoid of RNA Binding Activity. PLoS One 8: e64609.

6. Serganov A, Benard L, Portier C, Ennifar E, Garber M, et al. (2001) Role of conserved nucleotides in building the 16 S rRNA binding site for ribosomal protein S15. J Mol Biol 305: 785-803.

7. Fechter P, Chevalier C, Yusupova G, Yusupov M, Romby P, et al. (2009) Ribosomal initiation complexes probed by toeprinting and effect of trans-acting translational regulators in bacteria. Methods Mol Biol 540: 247-263.

8. Marzi S, Myasnikov AG, Serganov A, Ehresmann C, Romby P, et al. (2007) Structured mRNAs regulate translation initiation by binding to the platform of the ribosome. Cell 130: 1019-1031.

9. Plumbridge J, Soll D (1989) Characterization of cis-acting mutations which increase expression of a glnS-lacZ fusion in Escherichia coli. Mol Gen Genet 216: 113-119.

10. Bolivar F, Rodriguez RL, Betlach MC, Boyer HW (1977) Construction and characterization of new cloning vehicles. I. Ampicillin-resistant derivatives of the plasmid pMB9. Gene 2: 75-93.

11. Geissler S, Drummond M (1993) A counterselectable pACYC184-based lacZ alpha-complementing plasmid vector with novel multiple cloning sites; construction of chromosomal deletions in Klebsiella pneumoniae. Gene 136: 253-255.

12. Christiansen L, Pedersen S (1981) Cloning, restriction endonuclease mapping and post-transcriptional regulation of rpsA, the structural gene for ribosomal protein S1. Mol Gen Genet 181: 548-551.

13. Caillet J, Nogueira T, Masquida B, Winter F, Graffe M, et al. (2003) The modular structure of Escherichia coli threonyl-tRNA synthetase as both an enzyme and a regulator of gene expression. Mol Microbiol 47: 961-974.

14. Wower IK, Zwieb CW, Guven SA, Wower J (2000) Binding and cross-linking of tmRNA to ribosomal protein S1, on and off the Escherichia coli ribosome. EMBO J 19: 6612-6621.
